# Biliary epithelial cells are facultative liver stem cells during liver regeneration in adult zebrafish

**DOI:** 10.1172/jci.insight.163929

**Published:** 2023-01-10

**Authors:** Isaac M. Oderberg, Wolfram Goessling

**Affiliations:** 1 Division of Genetics, Brigham and Women’s Hospital, Harvard Medical School, Boston, Massachusetts, USA.; 2 Harvard Stem Cell Institute, Cambridge, Massachusetts USA.; 3 Dana-Farber Cancer Institute, Harvard Medical School, Boston, Massachusetts, USA.; 4 Broad Institute of MIT and Harvard, Cambridge, Massachusetts, USA.; 5 Harvard-MIT Division of Health Sciences and Technology, Boston, Massachusetts, USA.; 6 Division of Gastroenterology, Massachusetts General Hospital, Harvard Medical School, Boston, Massachusetts, USA

**Keywords:** Hepatology, Stem cells, Adult stem cells

## Abstract

The liver is a highly regenerative organ, yet the presence of a dedicated stem cell population remains controversial. Here, we interrogate a severe hepatocyte injury model in adult zebrafish to define that regeneration involves a stem cell population. After near-total hepatocyte ablation, single-cell transcriptomic and high-resolution imaging analyses throughout the entire regenerative timeline reveal that biliary epithelial cells undergo transcriptional and morphological changes to become hepatocytes. As a population, biliary epithelial cells give rise to both hepatocytes and biliary epithelial cells. Biliary epithelial cells proliferate and dedifferentiate to express hepatoblast transcription factors prior to hepatocyte differentiation. This process is characterized by increased MAPK, PI3K, and mTOR signaling, and chemical inhibition of these pathways impairs biliary epithelial cell proliferation and fate conversion. We conclude that, upon severe hepatocyte ablation in the adult liver, biliary epithelial cells act as facultative liver stem cells in an EGFR-PI3K-mTOR–dependent manner.

## Graphical abstract

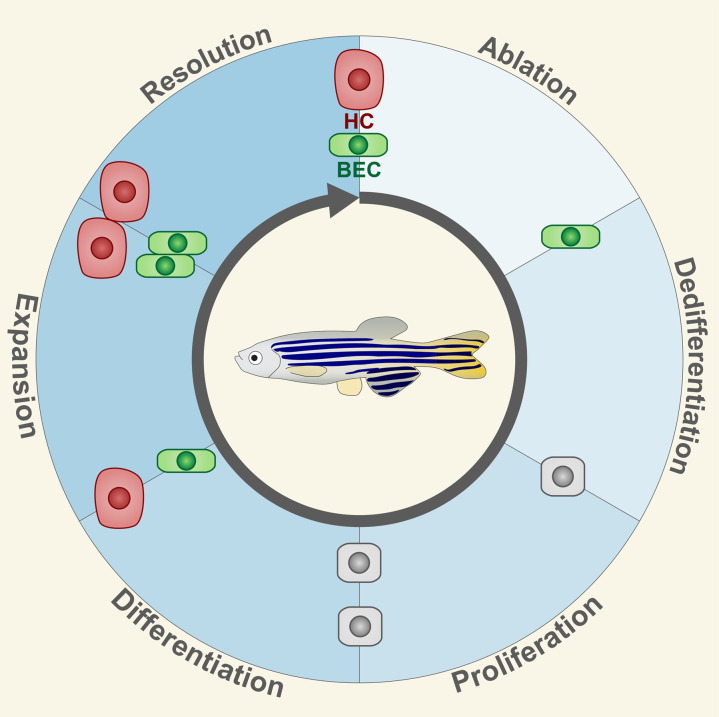

## Introduction

Regeneration is the replacement of cells or tissue lost to injury or damage (1); it is often mediated by a dedicated population of adult stem cells, defined by their ability to both self-renew and differentiate into multiple cell types. In humans, blood, skin, intestine, muscle, and airway epithelia all regenerate using tissue-specific adult stem cells (2). One notable exception is the liver, which is highly regenerative in response to injury, yet does not possess a universally defined adult stem cell. The liver performs vital functions for the organism, including secretion of blood coagulation factors, regulation of lipid and carbohydrate levels, xenobiotic detoxification, energy storage, and synthesis and secretion of bile and amino acids (3). The most abundant cells in the liver are hepatocytes, which carry out the core functions, whereas biliary epithelial cells (BECs) transport bile produced by hepatocytes to the gall bladder and intestine. During development, both hepatocytes and BECs are derived from bipotential endodermal cells called hepatoblasts (4). Other cell types in the liver include endothelial cells, hepatic stellate cells, portal fibroblasts, and a population of tissue-resident macrophages called Kupffer cells (5).

The zebrafish (*Danio rerio*) has emerged as a valuable model system for studying liver biology (6). Although tissue architecture is different from mammals (7), analysis of transgenic reporter lines during organ development revealed the cell type composition of the zebrafish liver, indicating the presence of hepatocytes (8), BECs (9, 10), endothelial cells (11), hepatic stellate cells (12), macrophages (13), and lymphocytes (14). An unbiased comparison between zebrafish and mammalian livers to determine conserved cellular markers and analogous function is missing, to date.

Both mice and zebrafish have been used to study cell fate transitions during liver regeneration. Elegant murine lineage-tracing studies have indicated that, during both homeostasis and regeneration from toxic or surgical injury, new hepatocytes arise from existing hepatocytes (15, 16). BECs are capable of contributing to hepatocyte regeneration in murine injury models only when hepatocyte proliferation is simultaneously inhibited (17). Consequently, there is currently no model involving a single insult to the adult liver to definitively determine and visualize the different steps involved to repair lost liver tissue. Moreover, these murine studies lack a molecular characterization of the presumed stem cell state. Zebrafish have been used to model liver regeneration after both toxic (18, 19) and surgical (20–25) liver injury, although cell fate transitions were not assayed in these models. In zebrafish larvae undergoing hepatocyte ablation, BECs can contribute to hepatocyte formation (26, 27). However, these studies were performed during development, and the observed potential of the BECs observed may be due to a lack of complete differentiation. It is also unclear whether the tissue microenvironment in the larval liver reflects that of an adult animal. Although many signaling pathways have been implicated in liver regeneration (28), not one has been definitively shown to regulate BEC-driven regeneration in adults. These studies raise the question of whether terminally differentiated BECs from adult zebrafish can act as facultative liver stem cells during regeneration and which signaling pathways regulate that process.

Here, we discover that regeneration of adult zebrafish liver can be achieved through a facultative stem cell model. We generated a complete cellular atlas of the uninjured, adult zebrafish liver using droplet-based single-cell RNA-Seq (scRNA-Seq). By comparing transcriptomic profiles of zebrafish liver cells to those of humans and mice, we reveal complete conservation of all cell types in zebrafish orthologous to their mammalian counterparts with preservation of the same functions. Analysis of multiple liver injury models in adults using genetic lineage tracing determined that BECs were only competent to give rise to hepatocytes after severe hepatocyte ablation. Sequencing BECs and their descendants after injury revealed a precise series of transcriptional changes, as BECs lose biliary markers and become morphologically and transcriptionally identical to hepatocytes. BECs proliferated and exhibited elevated levels of transcription factors associated with hepatoblast identity prior to their differentiation into hepatocytes. BECs showed activated MAPK, PI3K, and mTOR signaling during regeneration. Inhibition of EGFR, PI3K, or mTOR signaling resulted in defective regeneration. This work demonstrates that BECs can serve as facultative liver stem cells in adult zebrafish regenerating from severe hepatocyte ablation.

## Results

### A single-cell atlas for the adult zebrafish liver reveals high functional conservation with the mammalian liver.

**Figure 1:**
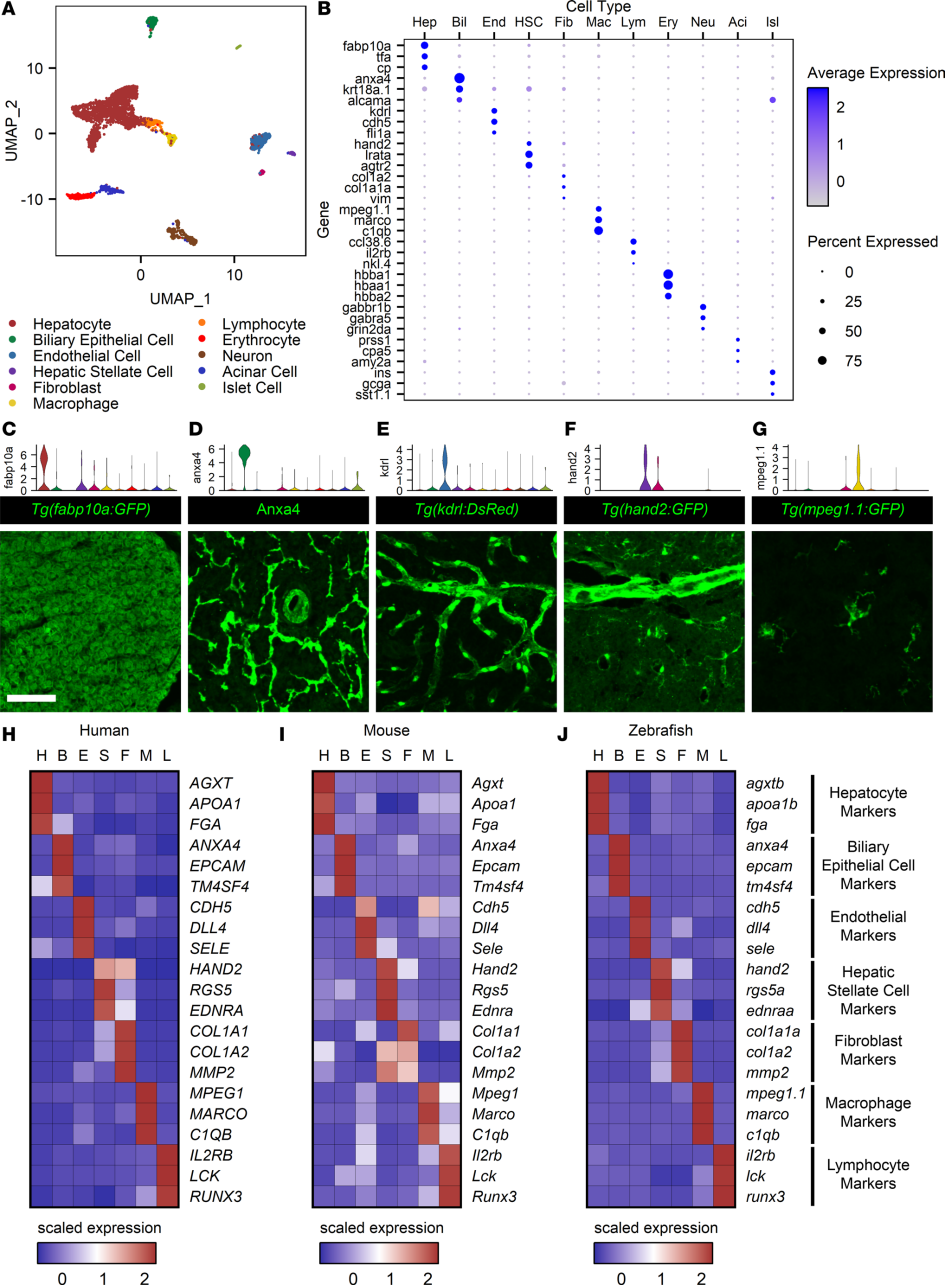
A single-cell atlas for the zebrafish liver. (**A**) UMAP plot showing 11 cell type clusters identified. (**B**) Dot plot for the 3 key markers used in cluster identification for each cell type cluster. Gene expression is represented by log-transformed normalized UMI counts, and the average expression is the mean of expression values for all cells in a given cluster. The color key from gray to blue indicates low to high expression levels, respectively. The size key indicates the fraction of cells in an individual cluster expressing a specific gene. (**C**–**G**) Paired violin plots and immunofluorescence (green) for *fabp10a* (**C**), *anxa4* (**D**), *kdrl* (**E**), *hand2* (**F**), and *mpeg1.1* (**G**). Scale bars: 50 μm. (**H**–**J**) Heatmaps displaying scaled expression for human (**H**), mouse (**I**), and zebrafish (**J**) orthologous genes. H, hepatocyte; B, biliary epithelial cell; E, endothelial cell; S, hepatic stellate cell; F, fibroblast; M, macrophage; and L, lymphocyte. Scaled expression values represent average expression values normalized to the minimum and maximum values in each row. The color key from blue to red indicates low to high scaled expression levels, respectively. Cell type markers are conserved across species.

To define the cell type repertoire of the liver, we performed scRNA-Seq using the Drop-Seq platform (29) on adult male zebrafish. A data set containing 3,148 cells enabled identification of 11 distinct clusters, which were assigned to a cell type based on known marker expression (Figure 1, A and B, and Supplemental Table 1; supplemental material available online with this article; https://doi.org/10.1172/jci.insight.163929DS1). Endodermal cell types in the liver, hepatocytes, and BECs were identified by the expression of *fabp10a*, *tfa*, and *cp* for hepatocytes (30–32) and *anxa4*, *krt18a.1*, and *alcama* for BECs (10, 33, 34). Clusters belonging to nonparenchymal cells in the liver were also identified: endothelial cells (*kdrl*, *cdh5*, *fli1a*; refs. 35–37), hepatic stellate cells (*hand2*, *lrata*, *agtr2*; refs. 12, 38), and a fibroblast population (*col1a2*, *col1a1a*, *vim*; ref. 39). Several clusters belonged to blood cells, including macrophages (*mpeg1.1*, *marco*, *c1qb*; refs. 40–42), lymphocytes (*ccl38.6*, *il2rb*, *nkl.4*; refs. 43), and erythrocytes (*hbba1*, *hbaa1*, *hbba2*; ref. 43), consistent with the mammalian liver. Cell types that represent contamination from other nearby organs were removed from downstream analyses. Expression of a subset of the genes identified by scRNA-Seq was confirmed in tissue sections via immunofluorescence (Figure 1, C–G). Visualization of hepatocytes via expression of *fabp10a* confirmed widespread distribution of hepatocytes in the liver (Figure 1C). Anxa4^+^ BECs formed a clear ductal network (Figure 1D). The *kdrl* expression highlighted the vascular architecture of the liver (Figure 1E). Interestingly, expression of the stellate cell marker *hand2* was detected both in the hepatic stellate cell and fibroblast clusters. Expression of *hand2* delineated both small cells inside of blood vessels and cells lining larger ducts (Figure 1F), suggesting that the fibroblast population may be portal fibroblasts. Finally, expression of *mpeg1.1* identified small cells with multiple protrusions (Figure 1G), as expected for a macrophage population. Publicly available data sets for the human (5, 42, 44) and mouse (45–47) liver were analyzed with a similar clustering scheme (Supplemental Figure 1, B and C). This revealed clusters corresponding to hepatocytes, BECs, endothelial cells, hepatic stellate cells, fibroblasts, macrophages, and lymphocytes, and it revealed markers that displayed tissue-specific expression across species for all of the cell types examined (Figure 1, H–J). The cell types with the most similar transcriptomes were hepatic stellate cells and fibroblasts, and this observation was true across species. These data reveal that the adult zebrafish liver has the same cellular inventory as the mammalian liver.

To better characterize these cell type clusters, we performed Gene Ontology (GO) analysis to identify terms overrepresented in each cluster (Supplemental Figure 1A and Supplemental Table 2). Hepatocytes perform many important biological functions, including hemostasis, lipid binding, oxidation reduction, amino acid metabolism, energy storage, and bile acid synthesis and transport. Hepatocyte-specific expression of genes associated with these functions was conserved across species (Supplemental Figure 1, D–F). Based on the well-described zonal architecture and heterogeneous transcriptional profile of mammalian livers (48), we sought to determine zebrafish hepatocyte heterogeneity in a separate analysis, revealing 2 main groupings of hepatocytes (Supplemental Figure 2A). Differential expression analysis (Supplemental Table 3 and Supplemental Figure 2E) indicated that one group was associated with the production and secretion of proteins (such as *tfa*; Supplemental Figure 2B) and the other was associated with metabolic enzymes (such as *tat* and *agxtb*; Supplemental Figure 2, C and D). We selected a handful of genes that were differentially expressed between the 2 clusters for further study (Supplemental Figure 2F). FISH revealed that expression of these genes was uniform throughout the liver, with no indication of spatially zonated gene expression (Supplemental Figure 2G). Taken together, this data set represents a single-cell atlas for the adult zebrafish liver, revealing that, while the tissue architecture of the liver is different, it is composed of cell types orthologous to mammalian liver. These cell types resemble their mammalian counterparts morphologically (Figure 1, C–G) and transcriptionally (Figure 1, H–J, and Supplemental Figure 1, D–F).

### BECs give rise to hepatocytes after massive hepatocyte ablation.

**Figure 2:**
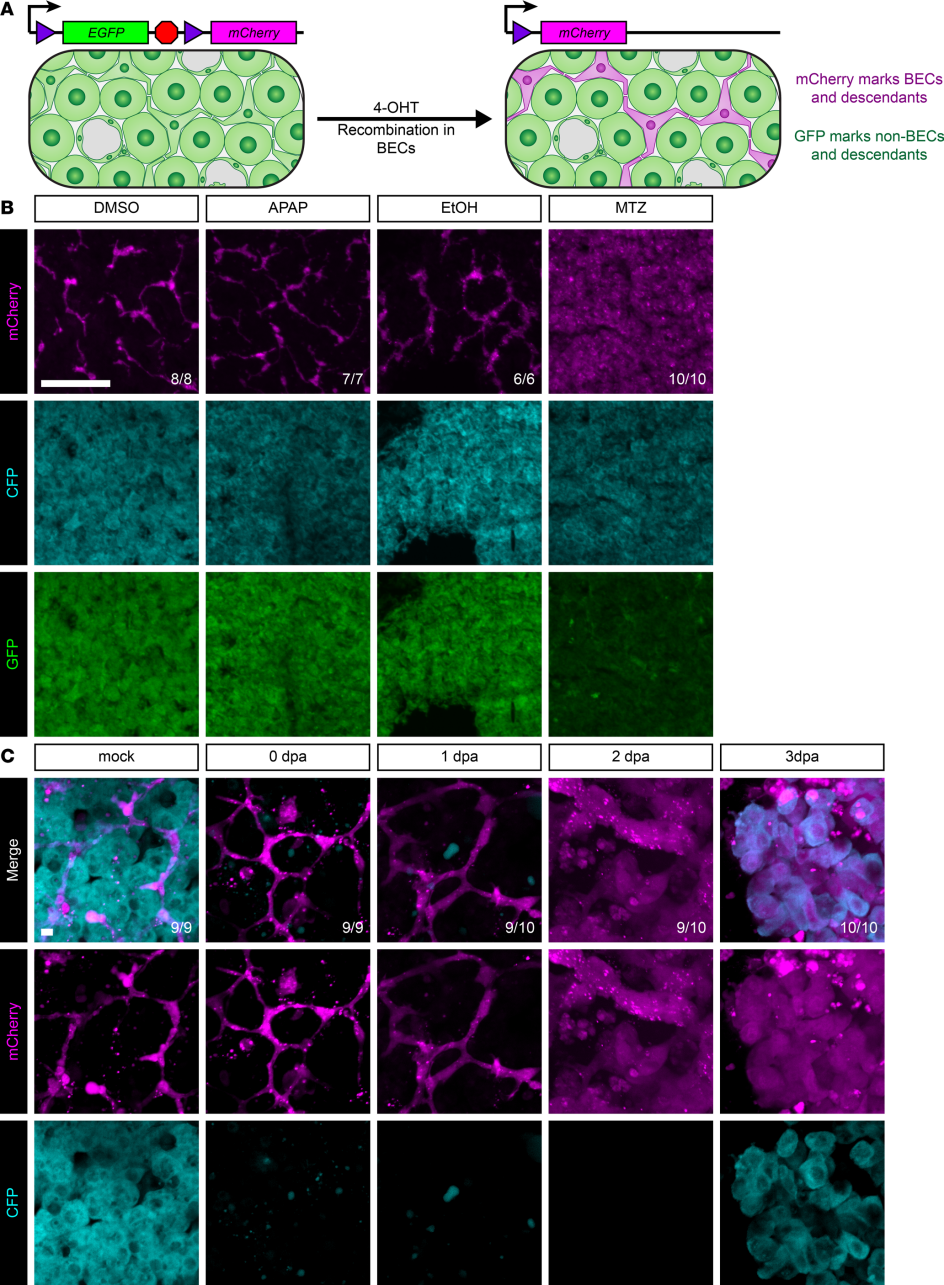
BECs give rise to hepatocytes only after hepatocyte ablation. (**A**) Schematic of recombination in triple-transgenic animals. Prior to 4-OHT administration, all cells in the liver are GFP^+^. After 4-OHT treatment, biliary epithelial cells are mCherry^+^. Because these transgenes are integrated into the genome, all descendants of mCherry^+^ cells will also be mCherry^+^. (**B**) Immunofluorescence showing mCherry (magenta, marking biliary and biliary-derived cells), GFP (green, marking hepatocyte-derived cells), and CFP (cyan, hepatocyte identity) signal in the adult liver for animals regenerating from various insults. DMSO, vehicle control (*n* = 8); APAP, acetaminophen (*n* = 7); EtOH, ethanol (*n* = 6); and MTZ, metronidazole (*n* = 10). Only after MTZ massive hepatocyte ablation do hepatocytes appear mCherry^+^, indicating biliary origin. Number of animals resembling the representative image are in white in the lower right corner of each image. Scale bars: 50 μm. (**C**) Live imaging time course of vibratome sections showing mCherry (magenta) and CFP (cyan) signal for adult livers regenerating after MTZ-induced hepatocyte ablation. Time points include mock (*n* = 9), 0 dpa (*n* = 9), 1 dpa (*n* = 10), 2 dpa (*n* = 10), and 3 dpa (*n* = 10). This demonstrates clear morphological changes in cells of biliary origin starting at 1 dpa and demonstrates that hepatocytes emerge within the mCherry^+^ lineage at 3 dpa. Number of animals resembling the representative image are in white in the lower right corner of each image. Scale bars: 5 μm.

Given the excellent molecular and cellular conservation between zebrafish and mammalian livers, we examined adult zebrafish livers to determine whether BECs could change their fate and become hepatocytes. To study lineage relationships during adult liver regeneration, we took advantage of 3 transgenic lines: a Cre driver, Cre responder, and a hepatocyte ablation line. *Tg(Tp1:CreERT2)* (49) contains Cre recombinase fused to an estrogen receptor under the control of a Notch-responsive element. Within the liver, Cre recombinase is exclusively expressed in BECs. The second line is the Cre-responder *Tg(ubi:Switch)* (50), a ubiquitous promoter driving GFP transgene expression. After recombination, the GFP transgene is deleted, and the ubiquitous promoter instead drives expression of mCherry (Figure 2A). Finally, we utilized the *Tg(fabp10a:CFP-NTR)* transgene (26), in which CFP fused to bacterial nitroreductase (NTR) is expressed specifically in hepatocytes. Exposure of animals to the nontoxic prodrug metronidazole causes cell death selectively in hepatocytes. Triple transgenic animals were exposed to the estrogen analog 4-hydroxytamoxifen (4-OHT) from 2 to 3 days postfertilization (dpf) to label BECs and their descendants, and treated zebrafish were raised to adulthood (Figure 2A). These adults were subjected to acetaminophen (APAP) or ethanol (EtOH) overdose (18, 51), partial hepatectomy (PHX) (25), or hepatocyte ablation via exposure to metronidazole (MTZ) (26). Regeneration was analyzed at 7 days posttreatment (dpt) following recovery at 28.5°C (Figure 2B). In DMSO or sham surgical controls, BECs were mCherry^+^ (biliary-derived), and hepatocytes were CFP^+^/GFP^+^ (nonbiliary-derived). After inducing liver injury with APAP, EtOH, or PHX, animals regenerating from these insults were identical to their respective controls — all hepatocytes were CFP^+^/GFP^+^ (nonbiliary-derived). Only severe hepatocyte ablation after MTZ exposure yielded mCherry^+^ hepatocytes derived from BECs (Figure 2B and Supplemental Figure 3A), demonstrating that only this extreme injury induces BECs to become hepatocytes.

These results prompted a detailed characterization of the process of regeneration following hepatocyte ablation. Triple transgenic animals were ablated and analyzed by imaging of live vibratome sections at 0, 1, 2, 3, and 7 days postablation (dpa) (Figure 2C and Supplemental Figure 3B). At 0 dpa, there was a dramatic reduction of CFP signal in the liver, consistent with massive hepatocyte ablation, whereas the biliary tree remained intact. Between 0 and 2 dpa, mCherry^+^ BECs changed in morphology and increased in number. By 3 dpa, CFP expression emerged in mCherry^+^ cells, and this persisted to 7 dpa (Figure 2C and Supplemental Figure 3B), indicating that mCherry^+^ BECs had become hepatocytes.

To quantify these cellular changes, we imaged cells isolated from the liver and used an automated image analysis pipeline to measure fluorescence intensity and morphological characteristics during regeneration (Supplemental Figure 4, A–D). We compared CFP intensity, cell area, and cell eccentricity between the GFP and mCherry lineages in uninjured animals. GFP^+^ cells from mock-treated animals (predominantly hepatocytes) have higher CFP intensity, are larger, and are more circular, with lower eccentricity than mCherry^+^ cells (Supplemental Figure 3, C–E). We examined these parameters over time in mCherry^+^ cells. CFP expression was not significantly different from mock-treated mCherry^+^ BECs until it increased at 3 dpa and continued to enhance at 7 dpa (Supplemental Figure 3F). mCherry^+^ cells were significantly larger at 2 and 7 dpa (Supplemental Figure 3G) and exhibited lower eccentricity only at 7 dpa (Supplemental Figure 3H). While there was a striking decrease in hepatocyte number after ablation, some GFP^+^ hepatocytes survived the ablation (Supplemental Figure 3B). We estimate that the proportion of hepatocytes that are derived from mCherry^+^ BECs ranges from 85% to 100% (Supplemental Figure 4E).

The presence of GFP^+^ hepatocytes at the terminal time point (Supplemental Figure 3B) suggested that these surviving hepatocytes contributed to regeneration. When comparing these hepatocyte-derived hepatocytes (HC) to biliary-derived hepatocytes (BDHC) at 7dpa, they were indistinguishable by CFP fluorescence (Supplemental Figure 3I) and cellular area (Supplemental Figure 3J). The BDHC were even more circular than the HC (Supplemental Figure 3K). These results indicate that BECs give rise to hepatocytes during regeneration from hepatocyte ablation.

### scRNA-Seq of biliary-mediated hepatocyte regeneration.

**Figure 3:**
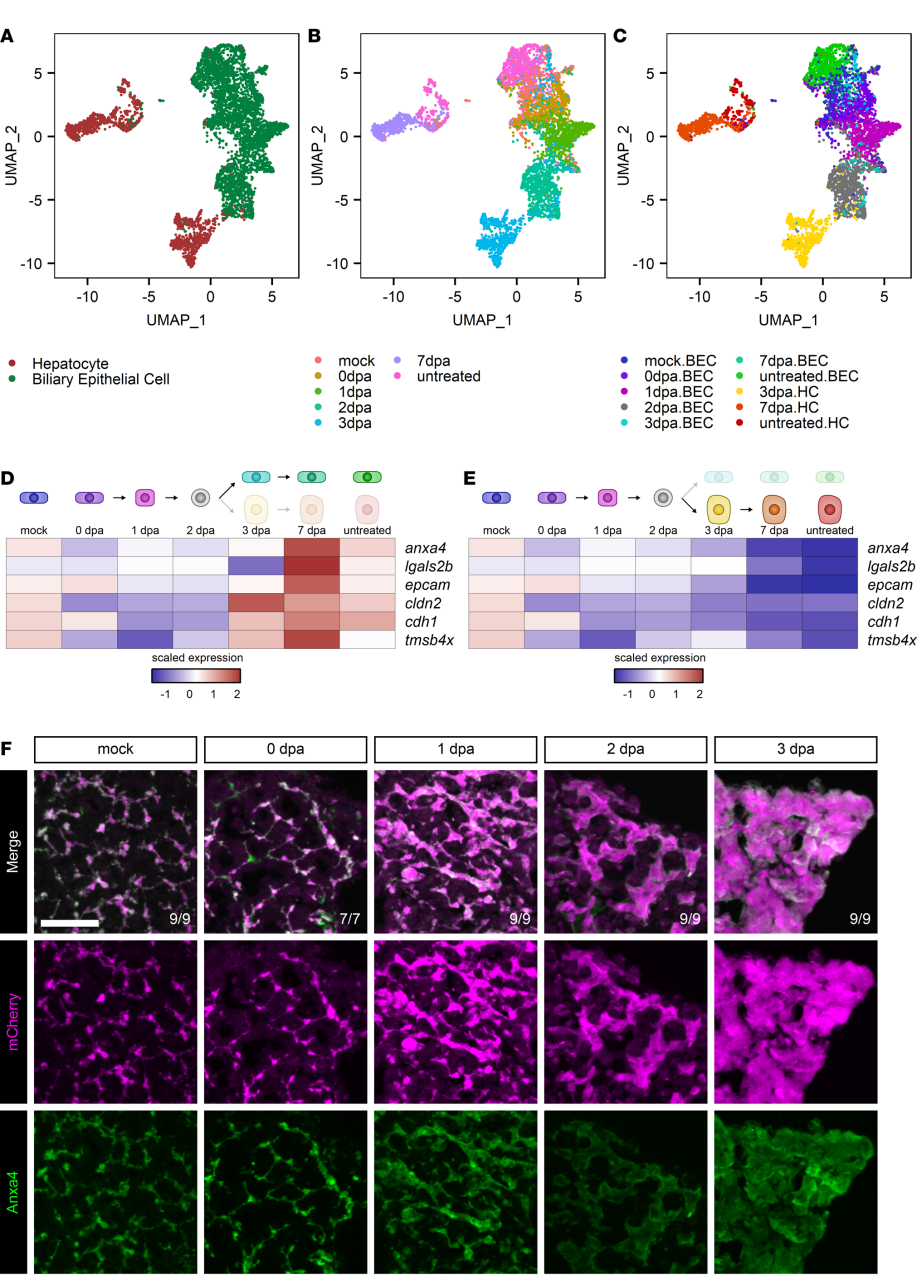
Single-cell sequencing of biliary-mediated regeneration. (**A**–**C**) UMAP plots of hepatocytes and BECs during regeneration from hepatocyte ablation. Cells are colored by cell type (**A**), time point (**B**), and cell state (**C**). (**D** and **E**) Heatmaps of the average expression in selected cell states for each gene. The scaled expression values are average expression values that have been normalized to the minimum and maximum values in each row. The color key from blue to red indicates low to high scaled expression levels, respectively. Data are shown for the biliary branch (**D**) and hepatocyte branch (**E**). Biliary markers initially decrease and then only return along the biliary branch. (**F**) Immunofluorescence showing mCherry (magenta) and Anxa4 (green) signal as markers for BEC origin and BEC identity, respectively, in adult livers in animals regenerating from hepatocyte ablation. Time points shown are mock (*n* = 9), 0 dpa (*n* = 7), 1 dpa (*n* = 9), 2 dpa (*n* = 9), and 3 dpa (*n* = 9). Number of animals resembling the representative image are in white in the lower right corner of each image. Scale bars: 50 μm.

To further define the molecular signals during regeneration after hepatocyte ablation, scRNA-Seq was performed at 0, 1, 2, 3, and 7 dpa. We used flow cytometry to positively select for mCherry^+^ cells to enrich for BECs and their descendants. After performing an initial round of clustering (Supplemental Figure 5A), clusters were isolated, corresponding to hepatocytes and BECs (Figure 3A). Because cells were isolated at known time points, we overlayed temporal information onto this plot (Figure 3B). We examined each time point in its own space (Supplemental Figure 5, B–H) and noted that, at 0–2 dpa, there were virtually no hepatocytes present. This persisted until 3 dpa, at which point a hepatocyte cluster reemerged (Supplemental Figure 5F). This cluster persisted at 7 dpa (Supplemental Figure 5G). We noted, in the untreated time point, 2 separate groups of hepatocytes (Supplemental Figure 5H). A comparison between these 2 groups revealed that they were highly similar, differing mostly by a group of genes that encode for vitellogenin proteins (Supplemental Table 6 and Supplemental Figure 5, I–K).

We combined the cell type and time information to make a representation of cell “state” (Figure 3C). Based on the cell state clusters we identified, it appeared that the BECs present after the ablation ultimately gave rise to new BECs and hepatocytes at 3 dpa. Thus, we hypothesized that the cells at 2 dpa represent a branch point in the cellular trajectory during differentiation, with one branch leading to BECs and the other branch leading to hepatocytes. This raised the interesting question of whether all BECs participate in the regenerative process. If some BECs retain their identity, while others dedifferentiate, we would expect to find a separate cluster of BECs that maintain their transcriptional state. We examined the expression of *anxa4* at the early stages of regeneration (Supplemental Figure 5, L–O) and did not find a separate *anxa4-*high cluster at 0, 1, or 2 dpa, suggesting that all BECs may participate in the regenerative process.

We performed differential gene expression analysis (Supplemental Table 4), comparing each time point with the preceding time point, followed by GO analysis (Supplemental Table 5 and Supplemental Figure 5P). At 0dpa.BEC (biliary epithelial cells at 0 dpa), there was enrichment for genes involved in rRNA processing and ribosome biogenesis. This was followed at 1dpa.BEC and 2dpa.BEC by translation and peptide biosynthetic processes. Only in the 3dpa.HC cluster did we observe terms associated with hepatocyte functions, such as small-molecule metabolic process, organic acid metabolic process, and carboxylic acid metabolic process.

Heatmaps to visualize average expression of genes associated with biliary identity in the biliary branch (Figure 3D) and the hepatocyte branch (Figure 3E) showed that biliary markers decreased initially and then returned only along the biliary branch. The gene *anxa4* decreased along the hepatocyte branch as regeneration proceeded (Figure 3E), and immunofluorescence of Anxa4 and mCherry during regeneration from hepatocyte ablation confirmed that Anxa4 protein levels decreased over time, yet it was still present at low levels at 7 dpa (Figure 3F and Supplemental Figure 6C). Given this observation, we can use either Anxa4 or mCherry to visualize BECs and their immediate descendants during regeneration. Examination of the kinetics of hepatocyte function recovery along the hepatocyte branch revealed that genes associated with hemostasis, lipid binding, oxidation reduction, amino acid metabolism, energy storage, and bile acid synthesis and transport were present at 3 dpa and later (Supplemental Figure 5Q). Expression of the *fabp10a-*driven CFP transgene and protein levels of Bhmt both returned at 3 dpa (Supplemental Figure 6, A and B). Therefore, mCherry^+^ BECs slowly lost biliary markers and ultimately produced new hepatocytes at 3 dpa.

### Clonal analysis demonstrates that BECs give rise to both BECs and hepatocytes during regeneration.

**Figure 4:**
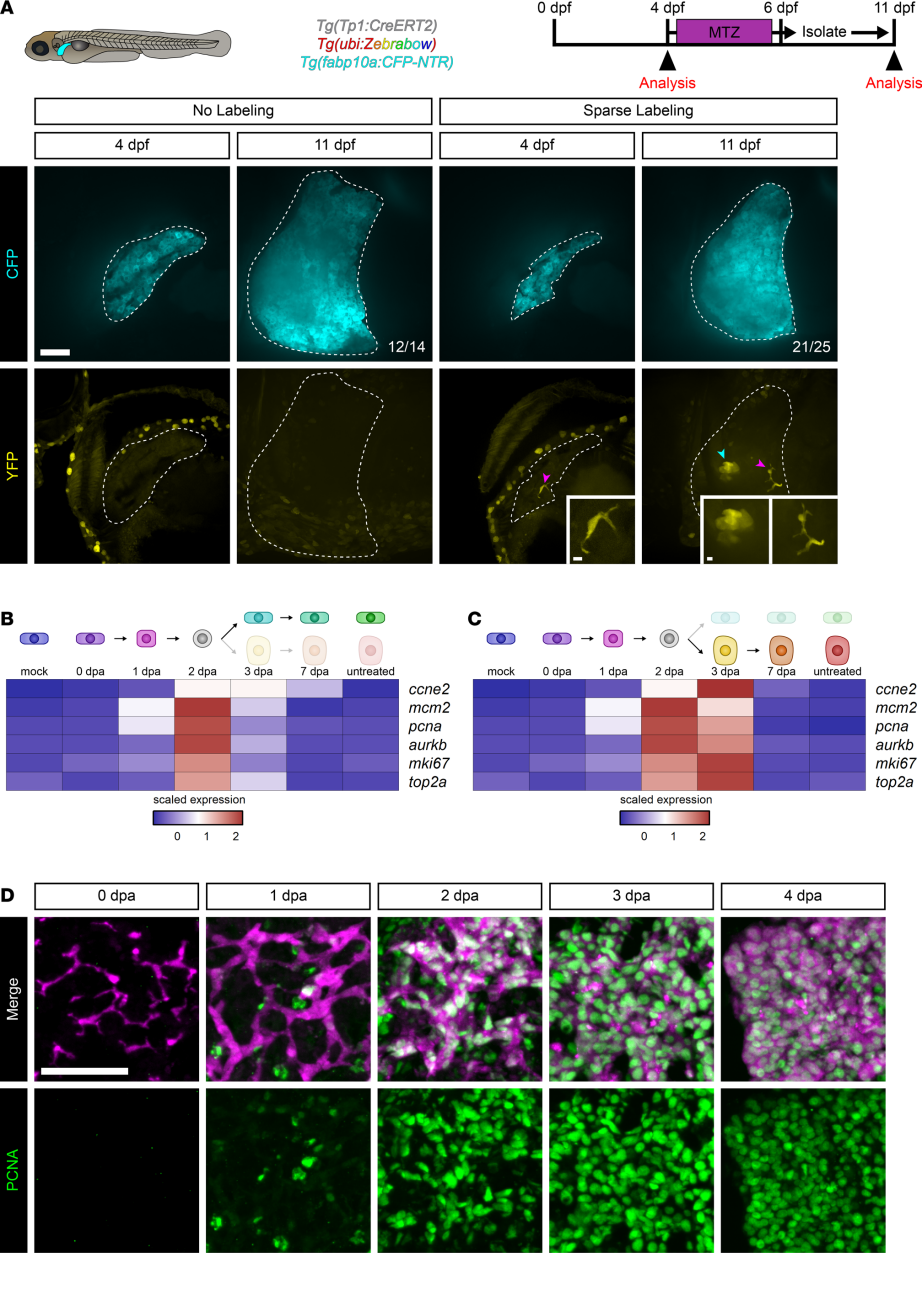
Clonal analysis of BECs during regeneration. (**A**) Live images of the zebrafish larval liver showing CFP (cyan) and YFP (yellow) signal for an individual animal with either no labeling (*n* = 14) or sparse labeling (*n* = 25) before and after ablation. Number of animals resembling the representative image are in white in the lower right corner of each image. White dotted line marks the boundary of the liver. Scale bars: 50 μm. Inset scale bars: 5 μm. Magenta arrowhead marks biliary epithelial cells, and cyan arrowhead marks hepatocytes. Limiting numbers of biliary epithelial cells give rise to colonies that have both biliary epithelial cells and hepatocytes. (**B** and **C**) Heatmaps of the average expression in selected cell states for each gene. The scaled expression values are average expression values that have been normalized to the minimum and maximum values in each row. The color key from blue to red indicates low to high scaled expression levels, respectively. Data are shown for the biliary branch (**B**) and hepatocyte branch (**C**). Markers of proliferation are highest at 2 and 3 dpa. (**D**) Immunofluorescence in adult liver showing mCherry (magenta) and PCNA (green) for animals regenerating from hepatocyte ablation. Time points shown are 0 dpa (*n* = 7), 1 dpa (*n* = 6), 2 dpa (*n* = 8), 3 dpa (*n* = 9), and 4 dpa (*n* = 5). There is a burst in proliferation ranging from 1 to 4 dpa. Scale bars: 50 μm.

What is the source of new BECs during regeneration from hepatocyte ablation? One explanation is that they also descended from the same BECs giving rise to hepatocytes. The population of mCherry^+^ cells isolated and sequenced at 7 dpa is comprised of both BECs and hepatocytes (Figures 3C and Supplemental Figure 5G), and at 7 dpa, there are small cells that are relatively high for Anxa4 protein that are also mCherry^+^ (Supplemental Figure 6C). An incidental observation was that when the *Tg(Tp1:CreERT2)* transgene was used in combination with *Tg(ubi:Switch)* or *Tg(ubi:Zebrabow)*, rare recombination events could occur in BECs even in the absence of 4-OHT induction, resulting in sparse labeling of individual BECs. We took advantage of this minimal reporter leakage to perform unprecedented clonal analysis in zebrafish larvae. Triple-transgenic animals (*Tg[Tp1:CreERT2]; Tg[ubi:Zebrabow]; Tg[fabp10a:CFP-NTR]*) were generated and deliberately not exposed to 4-OHT. Animals were screened by confocal microscopy at 4 dpf for those animals that had very few BECs labeled (1–4). These animals were isolated, subjected to hepatocyte ablation, allowed to recover, and imaged at 11 dpf. This approach allowed comparison of images at 4 and 11 dpf in the same animal. With high frequency, we observed that sparsely labeled BECs produced YFP^+^ colonies containing both BECs and hepatocytes (Figure 4A). Animals without labeling at 4 dpf also lacked labeling at 11 dpf with high frequency (Figure 4A), indicating that the presence of BECs as part of later colonies cannot be explained by leakiness after 4 dpf. Thus, as a population, BECs or their descendants are bipotent.

### mCherry^+^ BECs proliferate prior to hepatocyte differentiation.

**Figure 5:**
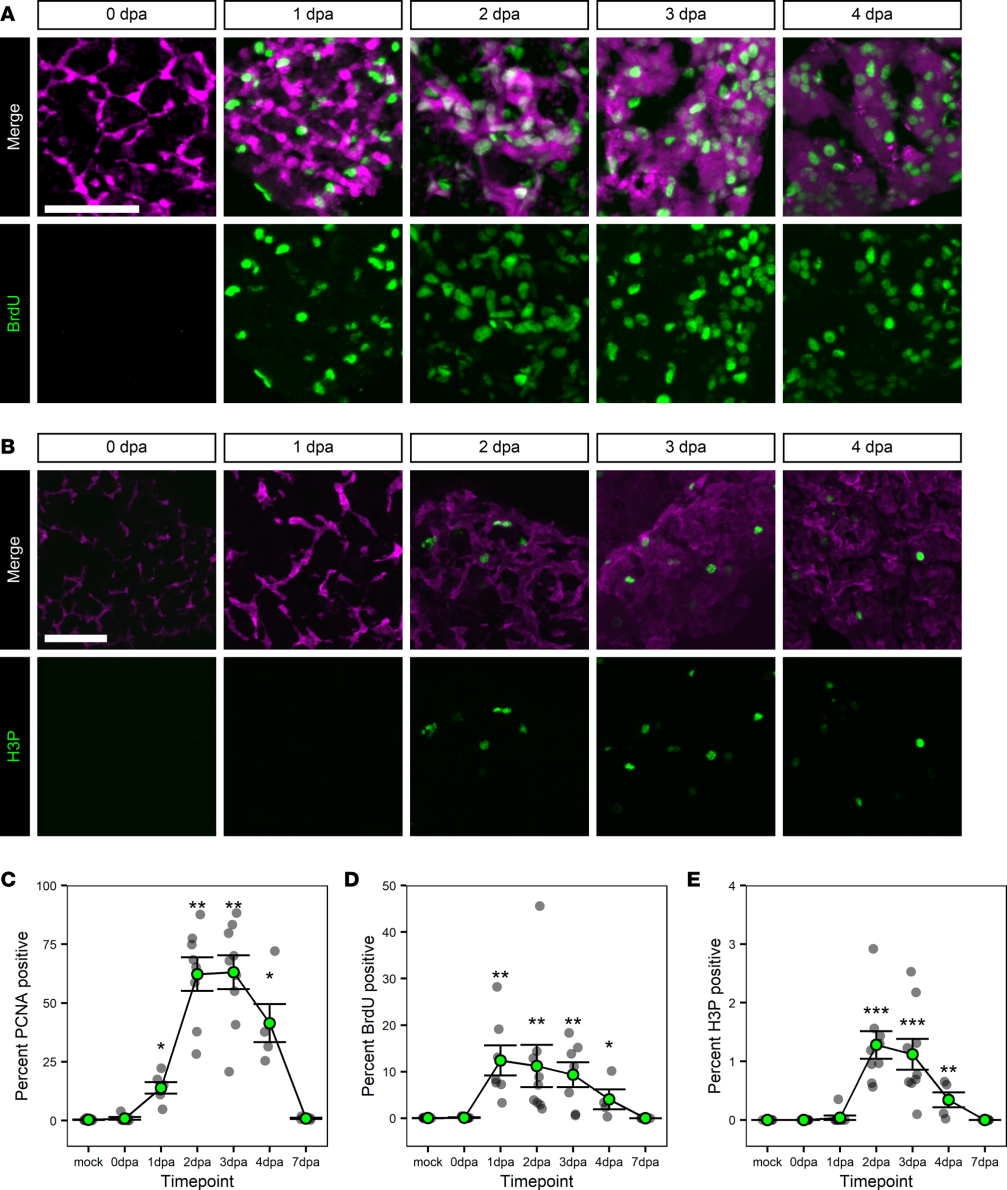
Proliferation of BECs during regeneration. (**A** and **B**) Immunofluorescence in adult liver showing markers of biliary origin (magenta) and proliferation/cell cycling (green); mCherry (magenta) and BrdU (green) (**A**) or Anxa4 (magenta) and H3P (green) (**B**) signal for animals regenerating from hepatocyte ablation. Time points shown are BrdU: 0 dpa (*n* = 7), 1 dpa (*n* = 7), 2 dpa (*n* = 9), 3 dpa (*n* = 7), and 4 dpa (*n* = 4); H3P: 0 dpa (*n* = 9), 1 dpa (*n* = 9), 2 dpa (*n* = 9), 3 dpa (*n* = 9), and 4 dpa (*n* = 5). There is a burst in proliferation ranging from 1 to 4 dpa. Scale bars: 50 μm. (**C**–**E**) Line graph of the percentages of nuclei positive for PCNA (**C**), BrdU (**D**), or H3P (**E**) over time. Gray dots mark the average value for an animal; green-filled dot marks the average of the animal values. Data are shown as mean ± SEM. For PCNA and BrdU, the earliest significant change as compared with mock sample occurs at 1 dpa. For H3P, the earliest significant change is at 2 dpa. Significance was determined using the Wilcoxon Rank Sum test, and *P* values were adjusted for multiple hypothesis testing using a Bonferroni correction. *P < 0.05, **P < 0.01, ***P < 0.001.

The observation that mCherry^+^ BECs can produce both more BECs and hepatocytes raises 2 possible models for this process, depending on whether differentiation or proliferation happens first. In the first case, a subset of BECs transdifferentiate into hepatocytes, after which the remaining BECs and hepatocytes proliferate to regenerate the liver. In the second model, BECs are capable of proliferating as they dedifferentiate, before differentiating into new BECs and hepatocytes. To distinguish between these models, markers for proliferation (52) in the scRNA-Seq data set along both cellular branches were examined (Figure 4, B and C). Highest expression of these genes was observed at 2 and 3 dpa, with some expression of the S-phase markers *mcm2* and *pcna* at 1 dpa. Immunofluorescence for the S-phase marker PCNA (Figure 4D and Supplemental Figure 7A) and BrdU incorporation in regenerating liver (Figure 5A and Supplemental Figure 7B) confirmed these observations. We also observed mitotic mCherry^+^ cells by labeling for phosphorylated histone H3 (H3P) between 2 and 4 dpa (Figure 5B and Supplemental Figure 7C). Quantification of nuclei in mCherry^+^ cells that were positive for PCNA (Figure 5C), BrdU (Figure 5D), or H3P (Figure 5E) revealed statistically significant increases in proliferation as early as 1 dpa, with proliferation having been terminated by 7 dpa. This timing, in conjunction with the observations that new hepatocytes differentiate at 3 dpa (Supplemental Figure 6, A and B), indicates that cell cycle reentry in mCherry^+^ cells precedes hepatocyte differentiation. These results indicate that BECs proliferate prior to differentiating into new BECs and hepatocytes.

Our prior observations about the proportion of hepatocytes that are mCherry^+^ between 3 dpa and 7 dpa (Supplemental Figure 4E) led us to hypothesize that mCherry^+^ hepatocytes have the same proliferative potential as GFP^+^ hepatocytes. To test this directly, we performed experiments with subtotal ablation. Treatment with 2.5 mM MTZ resulted in very little injury in most animals, although we noted that, in animals with small hepatocyte lesions, there was a local BEC response within the lesion (Supplemental Figure 8A). Treatment with 3.75 mM MTZ resulted in 7 of 13 animals with mosaic distribution of GFP^+^ and mCherry^+^ hepatocytes (Supplemental Figure 8B), and these GFP^+^ and mCherry^+^ cells had the same degree of proliferation (Supplemental Figure 8C). Finally, we performed an experiment in which animals were first subjected to a 5 mm MTZ ablation and were allowed to recover for 16 days, before being subjected to a second, 3.75 mM MTZ ablation (Supplemental Figure 9A). To our surprise, many of the animals recovering from ablation did not experience the same level of proliferation after a second injury (Supplemental Figure 9B). These results indicate that hepatocytes may need more time between injuries for their full proliferative potential.

### BECs express hepatoblast transcription factors during regeneration.

**Figure 6:**
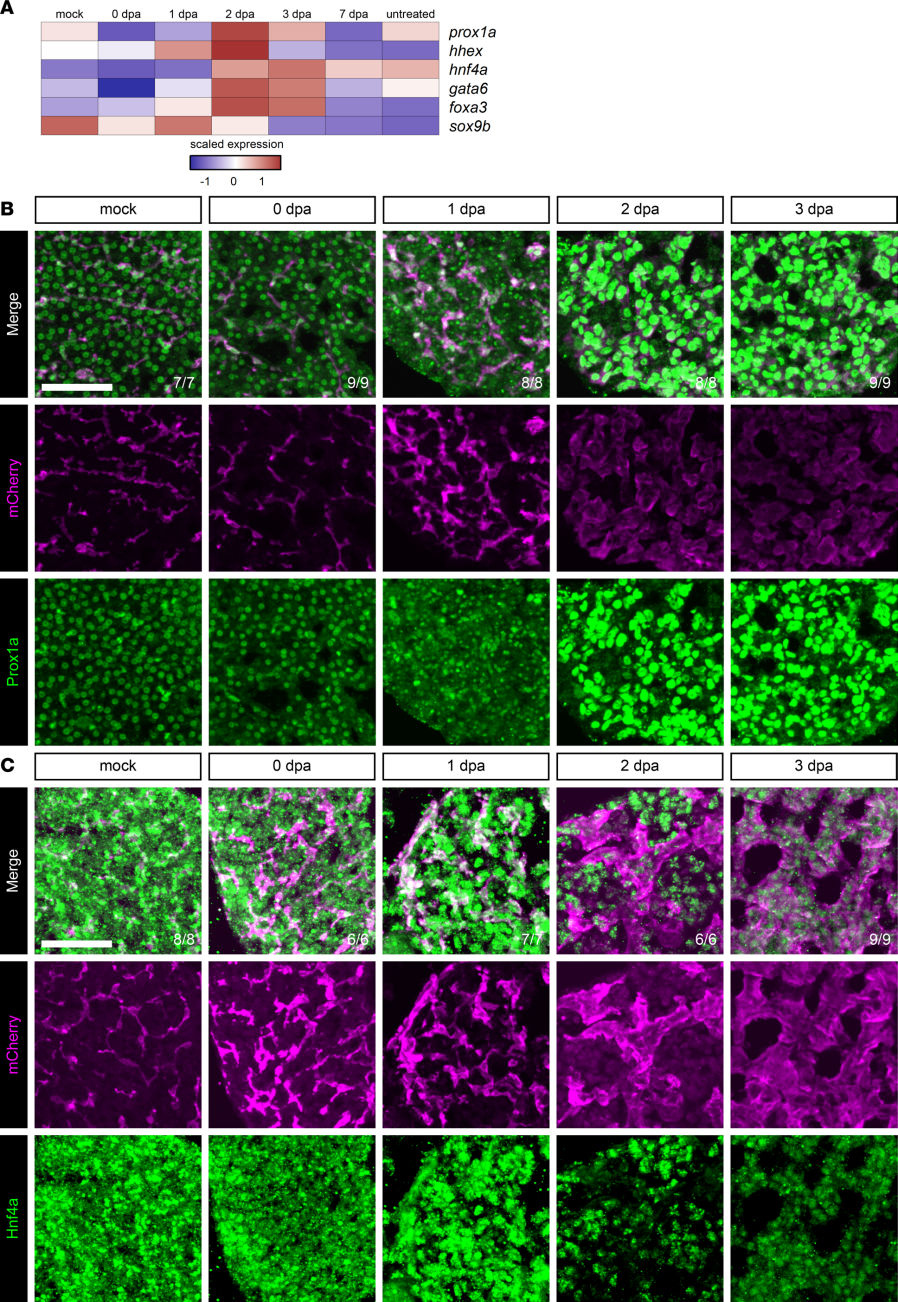
Transcription factor expression during regeneration from hepatocyte ablation. (**A**) Heatmap of the average expression in selected cell states for each gene along the hepatocyte branch. The scaled expression values are average expression values that have been normalized to the minimum and maximum values in each row. The color key from blue to red indicates low to high scaled expression levels, respectively. There is an elevation of transcription factor expression at 2 dpa. (**B** and **C**) Immunofluorescence showing Anxa4 (magenta) and Prox1a (green) or Hnf4a (green) signal in adult livers of animals regenerating from hepatocyte ablation. Time points shown are Prox1a: mock (*n* = 7), 0 dpa (*n* = 9), 1 dpa (*n* = 8), 2 dpa (*n* = 8), and 3 dpa (*n* = 9); Hnf4a: mock (*n* = 8), 0 dpa (*n* = 6), 1 dpa (*n* = 7), 2 dpa (*n* = 6), and 3 dpa (*n* = 9). Prox1a signal in Anxa4^+^ cells is elevated from 2 to 3 dpa. Nuclear Hnf4a signal is visible in Anxa4^+^ cells from 2 to 3 dpa. Number of animals resembling the representative image are in white in the lower right corner of each image. Scale bars: 50 μm.

During liver development, hepatoblasts are bipotent cells that produce both BECs and hepatocytes (4). We examined the expression of several transcription factors known to be expressed in hepatoblasts and required for normal liver development, including *prox1a*, *hhex*, *hnf4a*, *gata6*, *foxa3*, and *sox9b* (53). All these transcription factors, with the exception of *sox9b*, were elevated in BECs at 2 dpa compared with uninjured BECs (Figure 6A). Expression of Prox1a and Hnf4a by immunofluorescence (Figure 6, B and C, and Supplemental Figure 7, D and E) revealed Prox1a baseline expression in uninjured hepatocytes and BECs. Prox1a expression was elevated between 2 and 4 dpa, a timeframe that coincides with the onset of hepatocyte differentiation. We examined the protein levels of Hnf4a in Anxa4^+^ cells and found that a nuclear Hnf4a signal was visible at 2 dpa and subsequent time points. The presence of these transcription factors in Anxa4^+^ cells at 2 dpa prior to hepatocyte differentiation suggests that they could be acting to promote hepatocyte differentiation in regeneration, as they do in development.

### Growth factor signaling is elevated during liver regeneration.

**Figure 7:**
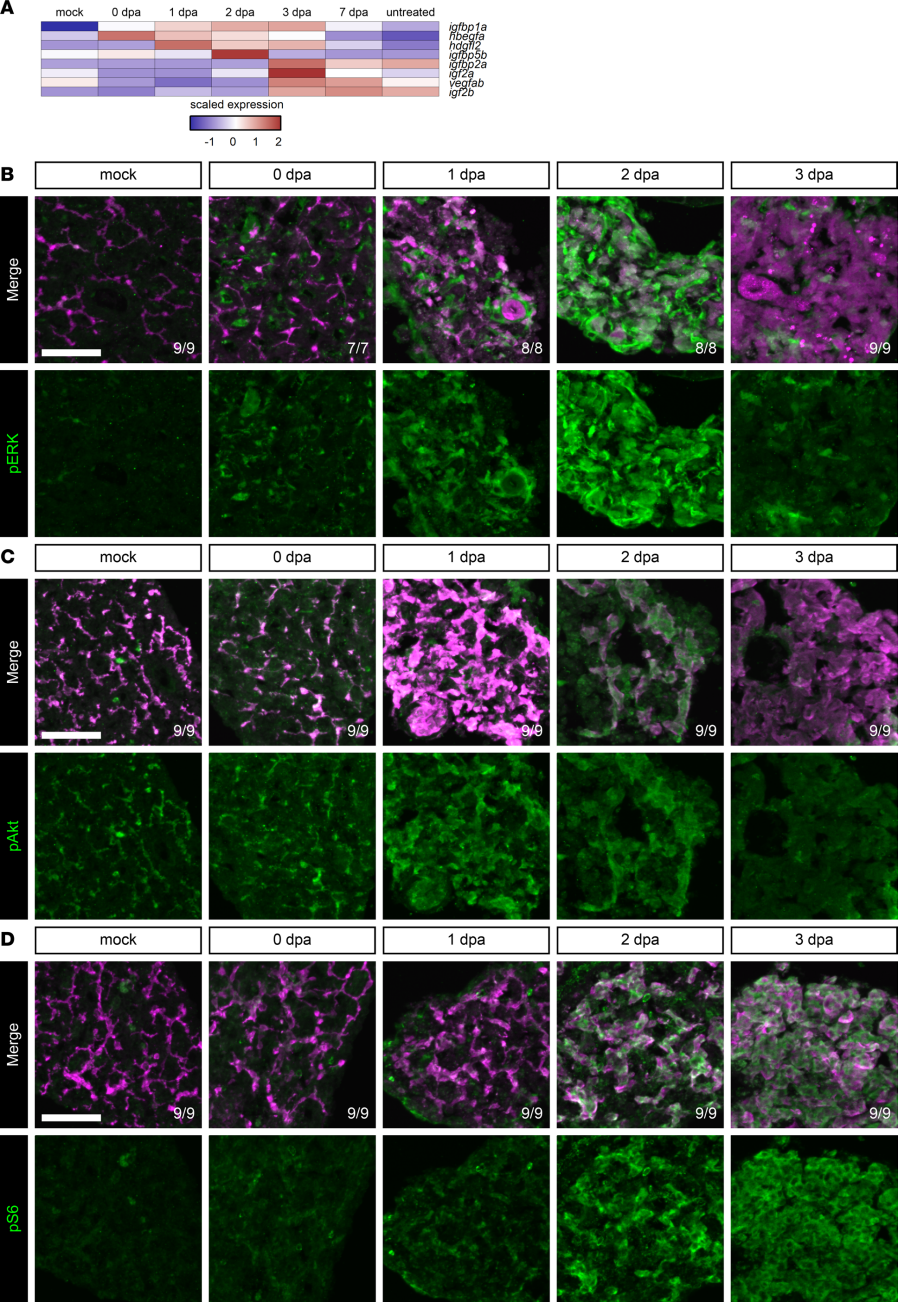
Growth factor signaling is elevated during regeneration from hepatocyte ablation. (**A**) Heatmap of the average expression in selected cell states for each gene along the hepatocyte branch. The scaled expression values are average expression values that have been normalized to the minimum and maximum values in each row. The color key from blue to red indicates low to high scaled expression levels, respectively. Two of the earliest growth factors elevated are *igfbp1a* and *hbegfa*. (**B**–**D**) Immunofluorescence in adult livers showing mCherry (magenta) and pERK (green) (**B**), Anxa4 (magenta) and pAkt (green) (**C**), or Anxa4 (magenta) and pS6 (green) (**D**) signal for animals regenerating from hepatocyte ablation. Time points shown are pERK: mock (*n* = 9), 0 dpa (*n* = 7), 1 dpa (*n* = 8), 2 dpa (*n* = 8), and 3 dpa (*n* = 9); pAkt: mock (*n* = 9), 0 dpa (*n* = 9), 1 dpa (*n* = 9), 2 dpa (*n* = 9), and 3 dpa (*n* = 9); and pS6: mock (*n* = 9), 0 dpa (*n* = 9), 1 dpa (*n* = 9), 2 dpa (*n* = 9), and 3 dpa (*n* = 9). MAPK, PI3K, and mTOR signaling are active during regeneration. Number of animals resembling the representative image are in white in the lower right corner of each image. Scale bars: 50 μm.

Growth factors play important roles in cell proliferation, differentiation, and survival (54). To identify potential growth factors regulating biliary-mediated liver regeneration after severe injury, growth factor ligands that were differentially expressed over time in the regeneration data set were identified (Figure 7A). One of the earliest ligands expressed was *hbegfa*, which has documented roles in adult neural regeneration in zebrafish (55). There was an induction of *hbegfa* expression in both BECs and neutrophils (Supplemental Figure 10, A–E), indicating that both autocrine and paracrine signaling mechanisms might be utilized during regeneration. Hbegfa can signal through EGFR, which has multiple downstream effectors including MAPK, PI3K-Akt, and mTOR signaling (56). To determine whether these pathways were elevated during regeneration, levels of pERK (Figure 7B and Supplemental Figure 10F), pAkt (Figure 7C and Supplemental Figure 10G), and pS6 (Figure 7D and Supplemental Figure 10H) were assayed. pERK signaling was increased between 1 and 2 dpa, indicating MEK activity during this window. pAkt signal was present in uninjured BECs and remained through 2 dpa, demonstrating a time window for active PI3K signaling. Finally, elevation of pS6 signal at 2 and 3 dpa revealed high mTOR activity. Taken together, MAPK, PI3K, and mTOR signaling pathways are active at 2 dpa, consistent with a role for these pathways in regulating regeneration.

### EGFR activation and downstream PI3K and mTOR activities are required for normal liver regeneration.

**Figure 8:**
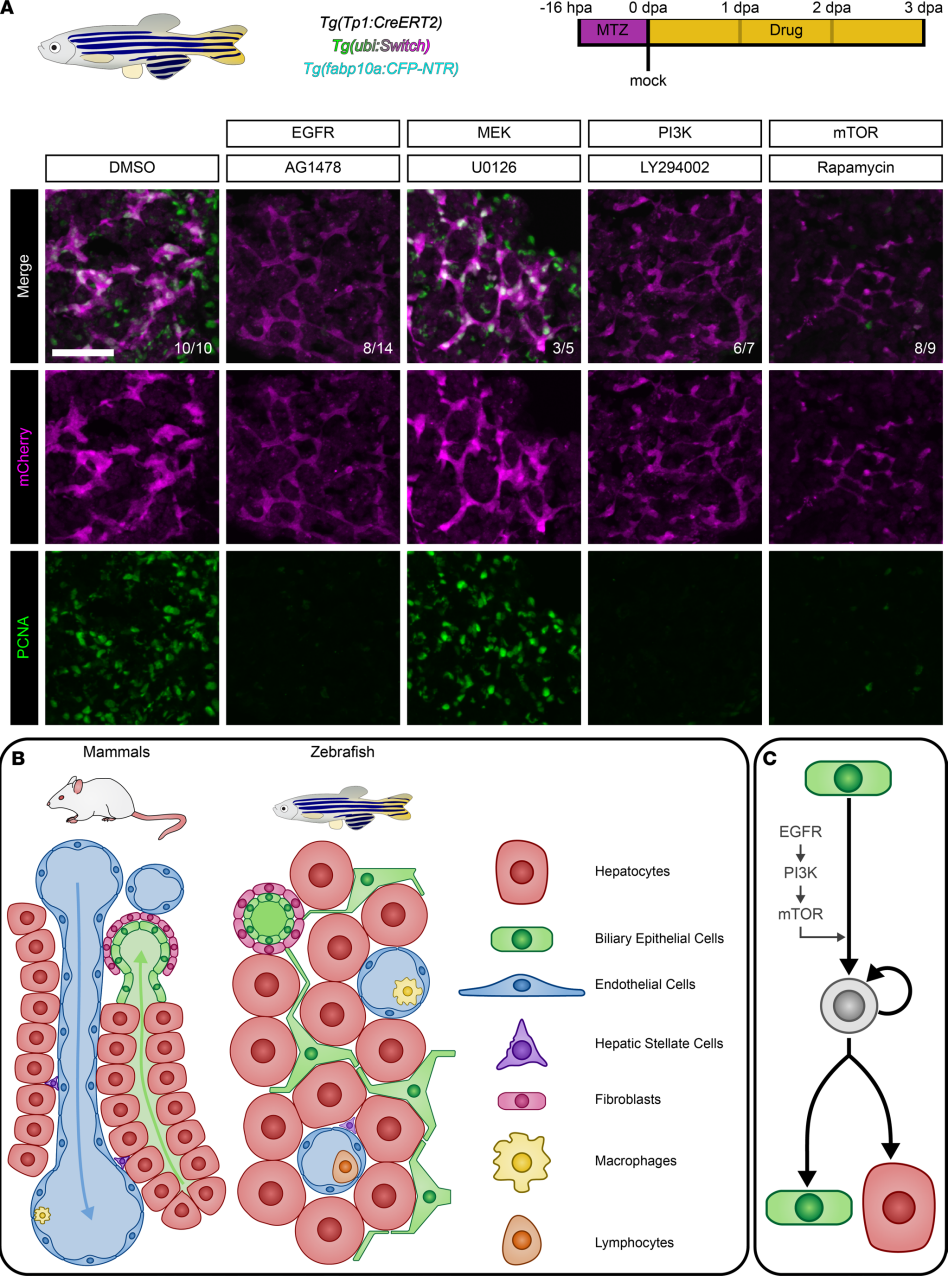
EGFR signaling is required for regeneration. (**A**) Immunofluorescence showing mCherry (magenta) and PCNA (green) signal for regenerating animals at 3 dpa.RT after chemical treatments. Inhibition of EGFR, PI3K, and mTOR dramatically reduces both cell proliferation and changes in cell morphology. Number of animals resembling the representative image are in white in the lower right corner of each image. Scale bars: 50 μm. (**B**) Illustration comparing mammalian and zebrafish liver architecture and cell types. (**C**) Model of biliary-mediated hepatocyte regeneration and the requirement for active EGFR, PI3K, and mTOR signaling.

To demonstrate a requirement for PI3K and mTOR signaling during normal liver regeneration, we employed a chemical inhibition strategy. To avoid accelerated degradation of chemicals, experiments with adult zebrafish were performed at room temperature (~21°C) rather than at 28°C, leading to an unexpected insight: at room temperature (Supplemental Figure 11A), regeneration proceeded in the same order but with slower kinetics. Under these conditions, the onset of major morphological changes and proliferation was at 3dpa.RT (3 dpa at room temperature), and reemergent hepatocyte gene expression occurred at 5dpa.RT. Based on this, we treated animals with inhibitors of EGFR (AG1478), MEK (U0126), PI3K (LY294002), and mTOR (rapamycin) from 0dpa.RT to 3dpa.RT. We observed both a decrease in BEC thickness and proliferation for animals treated with AG1478, LY294002, or rapamycin (Figure 8A and Supplemental Figure 13, A and B). Signaling pathway activity confirmed that the chemicals mechanistically functioned in zebrafish as expected (Supplemental Figure 12, A–C). AG1478 and U0126 both decreased levels of pERK, and AG1478 and LY294002 both decreased levels of pAkt. AG1478 and LY294002 caused a dramatic reduction in pS6, whereas rapamycin caused only a modest reduction. We repeated the experiment to determine the long-term consequences for animals exposed to these drugs during regeneration. Of the animals that were alive at 3dpa.RT, all (7 of 7) of the DMSO-treated animals survived to 19 dpa, whereas the survival rate was lower for animals subjected to AG1478 (4 of 10), LY294002 (4 of 12), and Rapamycin (2 of 10) exposure. This indicates that some of the animals with failed liver regeneration were never able to recover; those that managed to survive eventually regenerated (Supplemental Figure 14A).

Prior work in zebrafish larvae has suggested that, whereas inhibition of PI3K and mTOR blocked regeneration, inhibition of EGFR signaling might have a positive effect on regeneration (34, 57, 58). To confirm this, we performed drug experiments in zebrafish larvae (Supplemental Figure 13, C and D) and found our results in agreement with existing larval data, indicating that larval and adults may take advantage of different molecular mechanisms.

We performed the same chemical treatments with a later window from 2dpa.RT to 6dpa.RT to examine hepatocyte differentiation and proliferation (Supplemental Figure 15, A and B). Despite the mCherry^+^ cells still appearing to form a network, levels of proliferation or hepatocyte differentiation were not different between controls and drug-treated animals (Supplemental Figure 15, C–F), indicating that requirement for these signaling pathways occurs earlier during the regenerative process. These observations reveal that EGFR signaling through PI3K and mTOR, but not MAPK, is required for normal biliary-mediated regeneration following massive hepatocyte ablation.

## Discussion

In this study, we used scRNA-Seq combined with precise phenotyping to elucidate the molecular and cellular mechanisms underpinning liver regeneration in adult zebrafish. Our foundational characterization of uninjured zebrafish liver indicated morphological and transcriptional homology to mammalian models (Figure 8B), further motivating the use of this model for the study of liver regeneration. Lineage tracing of multiple injury models indicated that BECs change their fate and become hepatocytes only after massive hepatocyte ablation. Meticulous characterization of morphology, proliferation, and gene expression during this process indicates that BECs can act as facultative liver stem cells in an EGFR-PI3K-mTOR–dependent fashion (Figure 8C).

Our work establishes a cell type atlas for the adult zebrafish liver, containing all major cell types present in mammalian livers. This represents a definitive resource for zebrafish and liver research and is consistent with recent findings (59). It includes the first description, to our knowledge, of a portal fibroblast population in adult zebrafish liver, underscoring the advantage of an unbiased scRNA-Seq approach. Future studies can use zebrafish to study portal fibroblasts in their possible role in mediating biliary fibrosis (39). In mammals, hepatocytes are organized in hexagonal lobules, with a central vein at the center of each lobule and the portal veins, hepatic arteries, and bile ducts at each vertex. Hepatocyte functions tend to vary along this central-portal axis — a property termed zonation (60). Periportal hepatocytes are involved with the secretion of plasma proteins and clotting factors, whereas pericentral hepatocytes tend to carry out functions associated with detoxification, bile acid synthesis, and amino acid metabolism (61). Topologically, zebrafish also have portal and central veins, but their livers do not appear to have a lobular structure (25, 62). We describe 2 major groups of specialized hepatocytes, called Hep.1 and Hep.2, associated with different functions. Hep.1 hepatocytes were associated with GO terms such as peptide biosynthetic process, drug metabolic process, protein activation cascade, triglyceride homeostasis, and regulation of body fluid levels, similar to mammalian periportal hepatocytes. Hep.2 hepatocytes were associated with cellular amino acid metabolic process and fatty acid metabolic process, comparable with pericentral hepatocytes. Spatial analysis of gene expression revealed uniform expression of hepatocyte gene expression across the central-portal axis, in agreement with a previous report (62). Despite the architectural differences, the conservation between zebrafish and mammals makes the zebrafish an excellent model to study liver biology.

Liver regeneration has been studied for almost 100 years (63) using different model systems and modes of injury. The liver can regenerate via 1 of 2 modes: hepatocyte duplication or hepatocyte replacement from liver progenitor cells (LPCs). Importantly, although there is now significant evidence that the mode of liver regeneration is dependent upon the extent and type of injury (17), no definitive studies have been performed comparing single-injury models and following up with both detailed time-resolution and fate mapping. In mammalian models, new hepatocytes are generated from LPCs only when the injury is severe and additionally hepatocyte proliferation is compromised. These observations suggest the existence of an unknown molecular mechanism for sensing when hepatocyte regeneration has failed, triggering utilization of an alternate cellular source. Importantly, the precise identity of LPCs and whether LPCs are present in the uninjured liver remain controversial (64–69). The phenomenon of LPC-mediated regeneration was first observed in rats, where the process was termed ductular reaction, and the cells that ultimately give rise to hepatocytes were called oval cells (70). These putative LPCs are located in the canals of Hering, an anatomical structure that corresponds to the interface between hepatocyte canaliculi and bile ducts. The BECs in zebrafish are composed of 2 types, (a) intrahepatic BECs with intracellular lumens that form attachments with hepatocytes directly (71) and (b) extrahepatic BECs arranged into ducts that drain bile out of the liver. Given that intrahepatic BECs serve the same role and anatomical position as cells in the Canals of Hering, intrahepatic BECs may be the zebrafish equivalent of oval cells in rats. In addition to oval cells, LPCs have been given many different names, including hepatic progenitor cells (72), hepatic stem cells (73), hepatoblast-like cells (74), bipotential progenitor cells (58), and liver stem cells (75), highlighting the lack of definitive insight in the field. LPCs are defined primarily by their potential to generate both hepatocytes and BECs, rather than by their ability to proliferate (another important property of stem cells). Additionally, LPCs are sometimes defined by their blended transcriptional signature, in that they coexpress markers typically understood as distinctive markers of either hepatocytes or BECs. Here, we directly compared multiple-injury models in adult zebrafish using the same lineage-tracing tools and found that BECs contributed to regeneration only after extreme injury. Morphological and transcriptional observations of this process were made with unprecedented temporal resolution to identify the emergence of LPCs in adult liver regeneration. Zebrafish LPCs were capable of both self-renewal and differentiation into both hepatocytes and BECs, and they expressed transcription factors associated with hepatoblast identity. Thus, zebrafish LPCs have the capacity to act as facultative stem cells during regeneration following hepatocyte ablation.

Our observations after pharmacological inhibition of EGFR, PI3K, or mTOR signaling have prompted a proposed molecular mechanism for biliary-mediated hepatocyte regeneration. After hepatocyte ablation, BECs express *hbegfa* and signal to each other through EGFR in an autocrine fashion. This signal is transduced through the PI3K and mTOR pathways, promoting both proliferation in LPCs and changes in cell fate. In mammals, HBEGF is induced during multiple models of liver injury (76, 77), its loss negatively impacts regeneration (78), and it acts as a potent mitogen if exogenously introduced during regeneration (77, 79, 80). EGFR, PI3K, and mTOR have all been implicated in the proliferation of LPCs (81–84). Interestingly, our data on adult regeneration are consistent with data from larvae regarding the effect of PI3K (57) or mTOR (58) inhibition on biliary-mediated regeneration, but they are not consistent with data from larvae regarding the effect of EGFR inhibition (34). This observation indicates that larval and adult zebrafish employ different mechanisms during regeneration, and it further highlights the importance of performing regeneration experiments in adult animals.

The fact that EGFR signaling plays a role in regulating LPC proliferation is consistent with its history as a potent mitogenic pathway (85). What is more unusual, however, is that EGFR signaling was required for the dedifferentiation of BECs into LPCs. There are documented cases of EGFR signaling regulating dedifferentiation during regeneration from injury in the retina (55), heart (86), and kidney (87). In addition, mTOR signaling was found to be required both for dedicated quiescent stem cells to reenter the cell cycle (88) and for the creation of facultative stem cells during regeneration (89). Taken together with previous studies, our data indicate a significant role for EGFR and mTOR signaling in regulating facultative stem cell behavior. A currently unmet clinical need is the lack of cellular replacement therapy for patients with chronic liver disease and chronic liver failure, where the only available option is liver transplantation. Despite a severe reduction in the number of functional hepatocytes, hepatocyte proliferation is impaired. Targeted activation of EGFR and mTOR in BECs to convert this cell population to hepatocytes could represent a therapeutic avenue to treat these patients with advanced liver disease.

## Methods

A list of key reagents used in this study is included in Supplemental Table 8.

### Zebrafish.

Adult zebrafish (WT strains, Tübigen/TU and Tüpfel long fin/TL) and larva were raised at 28.5°C. Adult zebrafish were fasted for 24 hours prior to the start of any experiment. The following transgenic zebrafish lines were utilized: *Tg(-2.8fabp10a:EGFP)* (90), *Tg(-3.5ubb:LOXP-EGFP-LOXP-mCherry)* (referred to as *Tg[ubi:Switch]*) (50), *Tg(EPV.Tp1-Mmu.Hbb:Cre-ERT2*,*cryaa:mCherry)* (referred to as *Tg[Tp1:CreERT2]*) (91), *Tg(fabp10a:CFP-NTR)* (26), *Tg(kdrl:DsRed2)* (92), *Tg(mpeg1:EGFP)* (40), *Tg(ubb:LOX2272-LOXP-Tomato-LOX2272-Cerulean-LOXP-YFP)* (referred to as *Tg[ubi:Zebrabow]*) (93), and *TgBAC(hand2:EGFP)* (94). All adult experiments were performed on zebrafish between 6 months and 18 months of age. Unless otherwise noted, both male and female zebrafish were used for experiments.

### Surgery and dissection.

PHX was performed as previously described (25). Briefly, anesthetized zebrafish were placed ventral side up in the groove of a sponge. An abdominal incision was made using spring-loaded scissors. Forceps were used to separate the ventral lobe from the intestine. The ventral lobe was peeled away from the intestine and severed. Animals were allowed to recover for 7 days before their viscera were dissected and fixed. Dissection was performed by making an incision in the ventral body wall. The connections between the intestine and both the esophagus and anus were severed. The visceral organs were removed. For experiments that only utilized liver tissue, the liver was carefully peeled away from the rest of the visceral organs.

### BrdU injections.

BrdU (Cayman Chemical Company, 15580) was dissolved in Cortland’s Salt Solution at a concentration of 5 mg/mL. A glass Hamilton syringe was loaded with 10 μL of BrdU solution. Anesthetized zebrafish were placed ventral side up in the groove of a sponge. The syringe was used to inject the BrdU solution into the abdominal cavity. Animals were allowed to recover for 4 hours before their viscera were dissected and fixed.

### Chemical treatments.

MTZ (MilliporeSigma, M3761-25G) was dissolved in system water at a concentration of 5 mM and 0.5% DMSO. Adult zebrafish were exposed to MTZ for 16 hours at ambient temperature in the dark. APAP (MilliporeSigma, A7085-100G) was dissolved in system water at a concentration of 15 mM. EtOH (MilliporeSigma, EX0276-3) was dissolved in system water at a concentration of 1%. Adult zebrafish were exposed to APAP or EtOH for 24 hours at ambient temperature in the dark. Stock solutions for AG1478 (Selleck Chemicals, S2728), U0126 (Cayman Chemical Company, 70970), LY290002 (Selleck Chemicals, S1105), and Rapamycin (Selleck Chemicals, AY-22989) were made by dissolution in DMSO at a concentration of 10 μM, 20 μM, 10 μM, and 10 μM, respectively. These chemicals were diluted 1:1,000 in system water to make a working solution. Adult zebrafish were immersed in the working solution. Working solution was changed daily. 4-OHT (Hello Bio, HB2508) was dissolved in DMSO at a concentration of 10 mM, vortexed continuously for 15 minutes, and stored at –20°C. Prior to use, an aliquot of 4-OHT was incubated at 65°C for 10 minutes. Triple-transgenic larvae were exposed to 4-OHT between 2 and 3 days after fertilization to induce recombination.

### Tissue dissociation.

Isolated liver tissue was spaced in a microcentrifuge tube with 1 mL of PBS and 10 μL of Liberase stock solution (MilliporeSigma, 5401119001). Liberase stock solution was made by dissolution in water at a concentration of 2.5 mg/mL. The sample was incubated for 30 minutes at 37°C, with shaking at 600 rpm. The sample was pipetted repeatedly into a single-cell suspension. Cells were passed through a 40 μm mesh into a 5 mL polypropylene tube. A total of 3 mL of PBS was added to stop the dissociation. Samples were centrifuged at 300*g* for 5 minutes at 4°C. Supernatant was discarded. Sample was resuspended in 650 μL of PBS. A total of 150 μL of cell suspension was removed for control tubes, leaving 500 mL in the sample tube. Hoechst (Thermo Fisher Scientific, H3570) was added to the sample tube at a dilution of 1:1,000 and incubated for 45 minutes in the dark at ambient temperature. For experiments on WT zebrafish, Propidium Iodide (Thermo Fisher Scientific, P4170) was added to the sample tube at a dilution of 1:100 and incubated for 5 minutes in the dark at ambient temperature. For experiments on transgenic zebrafish, TOPRO-3 (Thermo Fisher Scientific, T3605) was added to the sample tube at a dilution of 1:10,000 and incubated for 5 minutes in the dark at ambient temperature.

### FACS.

Single-cell suspensions were sorted on a BD Aria Sorter with 85 µm nozzle and 35 psi pressure. For experiments on WT zebrafish, cells were collected that were Hoechst^+^ and Propidium Iodide^–^. For experiments on WT zebrafish, cells were collected that were Hoechst^+^ and TOPRO-3^–^, and also positive for either GFP or mCherry.

### scRNA-Seq library preparation and sequencing.

We performed Drop-Seq, as described (29). Briefly, cells were diluted to 160 cells/μL. Cell, beads, and oil were combined through a microfluidic channel to generate droplets. Droplets were broken, and beads were collected and washed. Beads were subjected to reverse transcription with Maxima H Minus Reverse Transcriptase. cDNA was amplified with KAPA HiFi HotStart ReadyMix. Amplified cDNA was made into libraries using the Nextera XT DNA Library Preparation Kit, with the goal of capturing 1,000 cells per library. Libraries were sequenced on an Illumina NextSeq 500 using the Single-End 75 bp kit with the following parameters: Read 1: 20 bp, Read 2: 50 bp, Read 1 Index: 8 bp, Custom Read 1 primer, 5′ - GCCTGTCCGCGGAAGCAGTGGTATCAACGCAGAGTAC - 3′.

### scRNA-Seq data preprocessing.

Fastq files were processed as described (29) using the 2.4.0 version of the Drop-Seq tools, mapping the reads to GRCz11 with the default parameters. For the DigitalExpression command, we set NUM_CORE_BARCODES = 5000. We used STAR version 2.5.3 and fastqc version 0.11.2. For each library, the barcodes were ranked in descending order by UMI counts. We generated a cumulative read fraction plot and calculated the slope at each point. The average slope was calculated. Each barcode below this average slope was classified as background, and each barcode above this slope was classified as a cell. For each gene, the distribution across all of the background barcodes was calculated, and the UMI count associated with 95% of the distribution was recorded. For each gene and each barcode, this UMI count was subtracted from the gene expression matrix, and any negative values were brought to zero. A new gene expression matrix was generated containing only the barcodes associated with cells for the background-subtracted data.

### scRNA-Seq data analysis.

Gene expression matrices were imported into Seurat version 4.0.3 with the parameters min.cells = 0, min.features = 0. Libraries were each imported into their own Seurat object, and they were then integrated using canonical correlation analysis. Gene expression data were normalized and scaled. Variable features were detected using the default parameters. Dimensionality reduction was performed using PCA and UMAP (no. of dimensions = 30). After an initial round of clustering, cells with no identifiable markers were removed. Variable feature detection, PCA, UMAP, and clustering were performed again. Clusters that were not different based on marker gene expression were merged. Cell type clusters were identified by the use of at least 3 markers per cluster. Differential gene expression was performed by calling FindAllMarkers (only.pos = T). GO enrichment analysis was performed using GOrilla (95) by comparing a list of differentially expressed genes against a background list of genes. We noted that 2 clusters corresponding to the major cell types in the pancreas were present in the data: acinar cells (*prss1*, *ela2l*, *cpa5*; ref. 96) and islet cells (*ins*, *gcga*, *sst1.1*; ref. 97) (Figure 1, A and B). These cells represent pancreatic contamination and are likely not present in the liver. Interestingly, one of the clusters expressed a number of neurotransmitter receptors (*gabbr1b*, *gabra5*, *grin2da*; ref. 98), suggesting that this cluster may represent a neuronal population (Figure 1, A and B). Glutamatergic enteric neurons have been identified in the intestine (99), so this population may be from intestinal contamination. These clusters were removed for downstream analyses. A similar analysis pipeline was used on the publicly available human and mouse data sets. Human data used in this paper are available under the accession nos. GSE124395, GSE115469, and GSE136103. Mouse data are available under the accession nos. GSE108097, GSE109774, and GSE132662. All graphs in this paper were generated using ggplot2 version 3.3.5. The genes appearing in figures are listed in Supplemental Table 7. Number of cells in each data set are reported in Supplemental Table 9. scRNA-Seq data generated in this paper are available from NCBI under the accession no. GSE217839.

### Rhodamine tyramide synthesis.

We dissolved 25 mg of NHS-rhodamine (5/6-carboxy-tetramethyl-rhodamine succinimidyl ester) in 2.5 mL of DMF and dissolved tyramine in 72 mM trimethylamine in DMF at a final concentration of 10 mg/mL. We added 744 μL of tyramine solution to the NHS-rhodamine solution and allowed the reaction to proceed in the dark for 2 hours. We stopped the reaction by adding 3.0 mL of EtOH, and we aliquoted and stored rhodamine tyramide at –80°C.

### Riboprobe synthesis.

For each gene of interest, a 1.5 kb region of cDNA including the ORF was cloned into pCR4-TOPO TA vector. PCR was used to amplify the cDNA region, and we added a T7 promoter to the 3′ end of the insert. T7 polymerase was used in an in vitro transcription reaction using a DIG RNA labeling mix to generate an antisense RNA probe (riboprobe).

### Immunostaining and FISH.

Zebrafish viscera were fixed by shaking in 4% paraformaldehyde in PBS for 24 hours at 4°C. Tissue was transitioned into 100% methanol and stored at –20°C for at least 24 hours. Tissue was then transitioned back to PBS, followed by 25% sucrose in PBS for 24 hours, 35% sucrose in PBS for 24 hours, and embedding in OCT and storage at –80°C. Blocks were sectioned on a Leica Cryostat CM3050 S. Slides were allowed to equilibrate for 20 minutes at ambient temperature. A hydrophobic barrier was drawn around the tissue. For immunostaining, samples were washed 4 times for 20 minutes in PBS+0.3% Tween-20. Samples were treated with Image-iT FX Signal Enhancer for 30 minutes, followed by blocking in 1% BSA and 5% normal goat serum for 1 hour. Samples were incubated in primary antibody overnight at 4°C. The next day, samples were washed in PBS+0.3% Tween-20 six times for 20 minutes each; they were then blocked again for an hour and incubated in secondary antibody overnight at 4°C. The next day, samples were washed in PBS+0.3% Tween-20 three times for 20 minutes each, incubated in a 1:5,000 DAPI solution for 1 hour, washed in PBS+0.3% Tween-20 three times for 10 minutes each, and mounted with Prolong Diamond Antifade Mountant (Thermo Fisher Scientific, P36961). For FISH, slides were thawed at room temperature for 20 minutes and a Secure Seal hybridization chamber was placed over the tissue. Samples were postfixed in 4% paraformaldehyde for 10 minutes, washed twice in PBS+0.3% Tween-20 for 5 minutes each, and treated with 20 ng/mL Proteinase K (Invitrogen, 25530-015). Enzymatic permeabilization was stopped by 15 minutes in 4% paraformaldehyde, followed by 4 washes PBS+0.3% Tween-20 for 5 minutes each. Samples were transitioned into prehybridization solution (PHYB) (50% deionized formamide, 5***×*** Saline-Sodium Citrate (SSC), 0.5 mg/mL yeast torula RNA, and 1% Tween-20) and incubated for 2 hours at 55°C. Subsequently, riboprobes were diluted in hybridization solution (HYB5D) (50% deionized formamide, 5***×*** SSC, 0.5 mg/mL yeast torula RNA, 0.1% Tween-20, 50 μg/mL heparin, and 5% dextran sulfate) at a concentration of 2 ng/μL, incubated for 5 minutes at 72°C, and added to slides. Riboprobes were hybridized for 16 hours at 55°C. The next day, samples were washed twice for 30 minutes at 55°C in each of the following solutions: PHYB, a 1:1 mixture of PHYB and 2× SSC 0.1% Tween-20, 2× SSC 0.1% Tween-20, and 0.2× SSC 0.1% Tween-20. After cooling to room temperature, samples were washed twice in PBS+0.3% Tween-20 for 10 minutes each and blocked in 20% lamb serum and 2% BMB (Blocking Reagent, MilliporeSigma) for 1 hour. Anti–DIG-POD was added at a concentration of 1:500, and samples were incubated overnight at 4°C. The next day, samples were washed 7 times for 10 minutes each in PBS+0.3% Tween-20 and were then incubated in TSA Buffer (2M NaCl, 100 mM Boric acid) for 10 minutes. Tyramide signal amplification was performed by adding Tyramide working solution (1:1,000 rhodamine tyramide, 0.003% H_2_O_2_, 20 μg/mL 4-iodophenylboronic acid) to samples for 30 minutes. Samples were washed in PBS + 0.3% Tween-20 three times for 20 minutes each, incubated in a 1:5,000 DAPI solution for 1 hour, washed in PBS + 0.3% Tween-20 three times for 10 minutes each, and mounted with Prolong Diamond Antifade Mountant. Slides were incubated for 24 hours in the dark at ambient temperature, and they were subsequently stored at 4°C. Anti-GFP (Aves Labs, GFP-1020) and anti-DsRed (Takara Bio, 632496) were used at 1:500. All other primary antibodies were used at 1:100. All secondary antibodies were used at 1:200.

### Imaging.

Microscopy was performed using an inverted Ti2 (Nikon) microscope equipped with a Yokogawa CSUW1 spinning disc confocal unit, a CFI Apo LWD Lambda S 40XC WI (1.15 NA) objective lens (Nikon), and a Zyla 4.2 PLUS sCMOS camera (ANDOR). When imaging multiple time points for the same set of antibodies, laser strength, gain settings, and exposure time were kept constant.

### Quantitative image analysis.

The nd2 files generated by microscopy were processed with a custom script in FIJI (100) to perform Z-projection and set channel colors. A second script was used to generate representative image panels for figures. Quantification of percentage of nuclei positive for either PCNA, BrdU, or H3P was performed in Imaris. Imaris was also used to estimate mean filament diameter as a proxy for biliary thickness. For analysis of dissociated cell images, the R package EBImage version 4.36.0 was used. The GFP and mCherry channels were used to create a cellular mask, and the DAPI channel was used to create a nuclear mask. The centroids of all cells that had exactly 1 nucleus were identified, and a small image of each cell was saved into a new file in grid format. For each grid file, the cellular and nuclear masks were used to make both morphological and fluorescence measurements for each cell. For each data point, another script could export the image associated with that cell.

### Statistics.

To aid in deciding whether a gene was differentially expressed in one cell population relative to another, FindAllMarkers was used for the Wilcoxon Rank Sum test. A gene was considered differentially expressed if it had a *P*_adj_ < 0.05. For comparing means between 2 samples, the Wilcoxon Rank Sum test was also used. Two means were considered statistically significantly different with a *P* < 0.05. For GO analysis, only GO terms with a *P* < 0.001 were returned.

### Study approval.

Zebrafish (WT strains, Tübigen/TU and Tüpfel long fin/TL) were maintained according to IACUC (IACUC-BIDMC #506-2015) protocols.

## Author contributions

IMO and WG secured funding for the project. IMO and WG conceived the project and designed the experiments. IMO generated, analyzed, and validated the data. IMO and WG wrote and revised the manuscript.

## Supplementary Material

Supplemental table 1

Supplemental table 2

Supplemental table 3

Supplemental table 4

Supplemental table 5

Supplemental table 6

Supplemental table 7

Supplemental table 8

Supplemental table 9

Supplemental data

## Version 1 01/10/2023

Electronic publication
