# Supplementary material for: Biliary epithelial cells are facultative liver stem cells during liver regeneration in adult zebrafish
Source: JCI Insight. 2023 Jan 10;8(1):e163929. doi: 10.1172/jci.insight.163929 (PMC9870093; doi:10.1172/jci.insight.163929)
Supplement: Supplemental data [file jciinsight-8-163929-s201.pdf]

Supplemental Material

Supplemental Figure 1: Related to Figure 1

Supplemental Figure 2: Related to Figure 1

Supplemental Figure 3: Related to Figure 2

Supplemental Figure 4: Related to Figure 2

Supplemental Figure 5: Related to Figure 3

Supplemental Figure 6: Related to Figure 3

Supplemental Figure 7: Related to Figures 4, 5 and 6

Supplemental Figure 8: Related to Figures 4 and 5

Supplemental Figure 9: Related to Figures 4 and 5

Supplemental Figure 10: Related to Figure 7

Supplemental Figure 11: Related to Figure 8

Supplemental Figure 12: Related to Figure 8

Supplemental Figure 13: Related to Figure 8

Supplemental Figure 14: Related to Figure 8

Supplemental Figure 15: Related to Figure 8

Supplemental Figure 1. Related to Figure 1

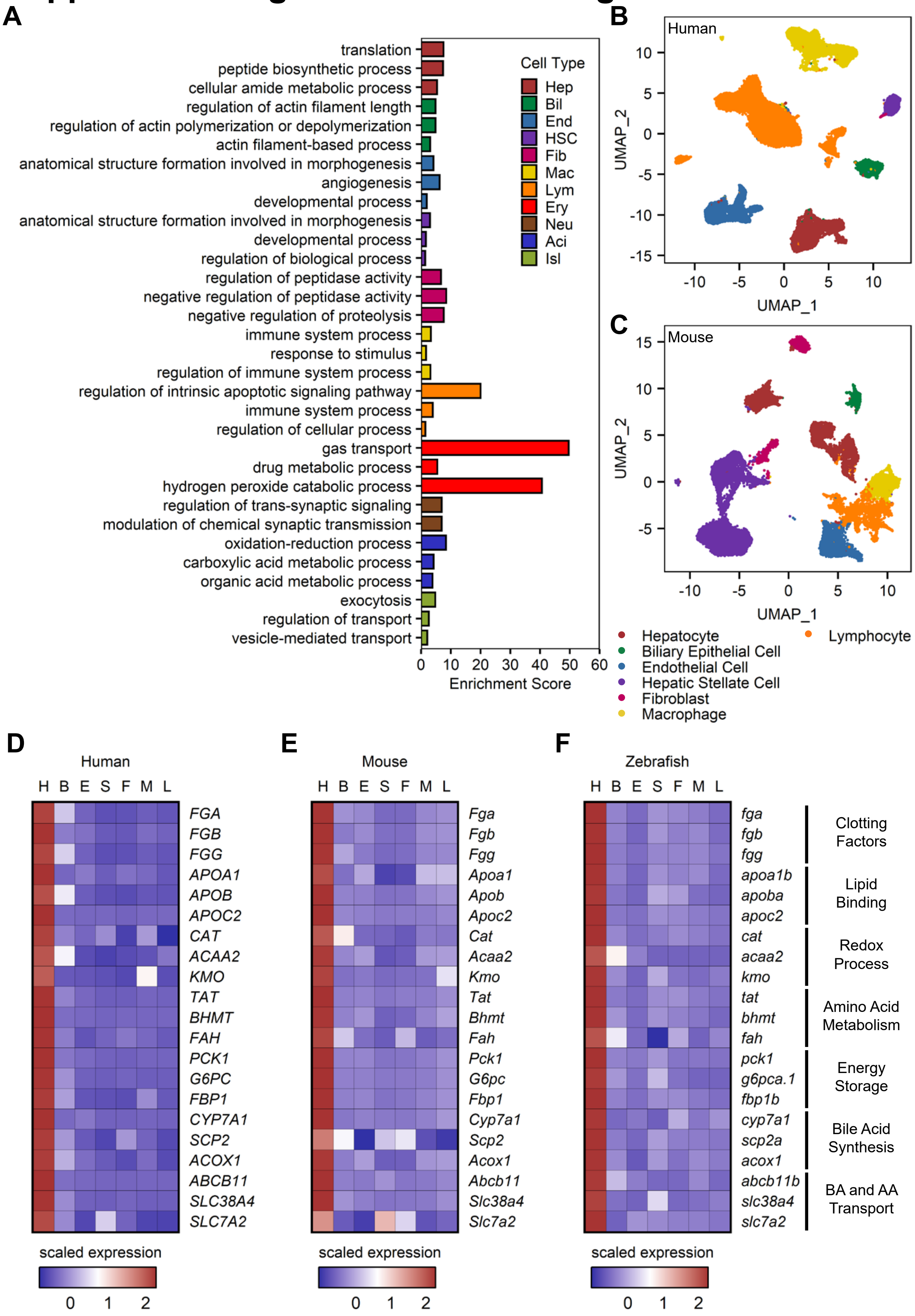

Supplemental Figure 1: Related to Figure 1

(A) Bar graph for the top three GO terms for each cell-type cluster from scRNA-seq of normal adult zebrafish liver. The length of the bar is the level of enrichment for that term in that cluster. The bars are colored by cluster. Hep=hepatocyte, Bil=biliary epithelial cell, End=endothelial cell, HSC= hepatic stellate cell, Fib=fibroblast, Mac=macrophage, Lym=lymphocyte, Ery=erythrocyte, Neu=neuron, Aci=acinar cell, and Isl=islet cell.

(B-C) UMAP plot for the publicly available clustered (B) human and (C) mouse data.

(D-F) Heatmaps displaying average expression for (D) human, (E) mouse, and (F) zebrafish orthologous genes. H=hepatocyte, B=biliary epithelial cell, E=endothelial cell, S= hepatic stellate cell, F=fibroblast, M=macrophage, and L=lymphocyte. The functions associated with the genes is written on the right. The scaled expression values are average expression values that have been normalized to the minimum and maximum values in each row. The color key from blue to red indicates low to high scaled expression levels, respectively.

Supplemental Figure 2. Related to Figure 1

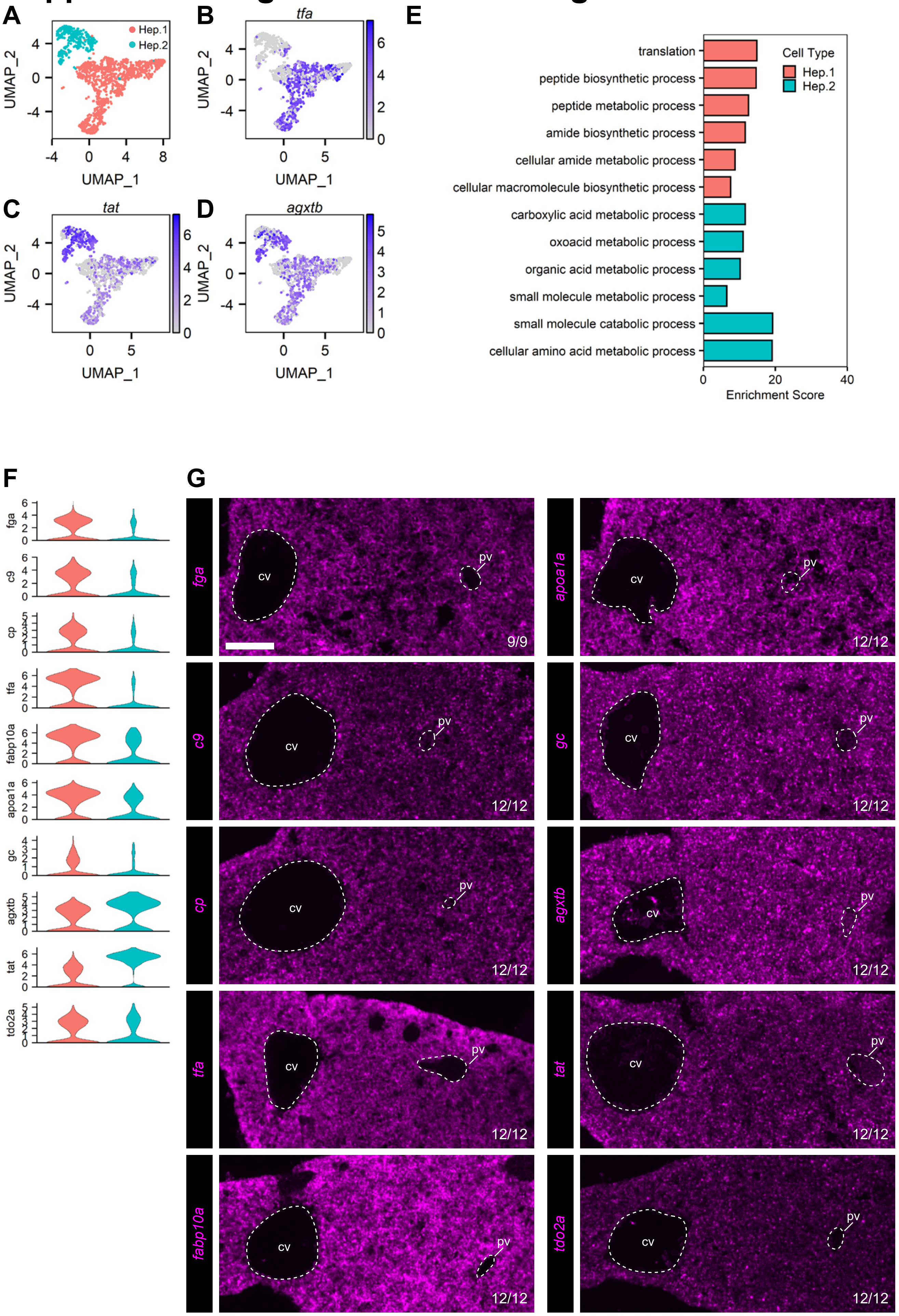

Supplemental Figure 2: Related to Figure 1

(A) UMAP plots of the hepatocytes from uninjured animals. Two clusters (Hep.1, red, Hep.2, cyan) are shown to demonstrate hepatocyte heterogeneity.

(B-D) Feature plots on hepatocytes from uninjured animals for (B) *tfa*, (C) *tat*, and (D) *agxtb*. The color key from gray to blue indicates low to high expression levels, respectively.

(E) Bar graph for the top six GO terms for each hepatocyte cluster. The length of the bar is the level of enrichment for that term in that cluster. The bars are colored by cluster. Hep.1 and Hep.2 hepatocyte clusters are associated with different functions.

(F) Violin plots for ten genes displaying differential expression between Hep.1 and Hep.2. (Hep.1=red, Hep.2=cyan).

(G) Fluorescent *in situ* hybridization for showing mRNA expression (in magenta) for *fga*, *c9*, *cp*, *tfa*, *fabp10a*, *apoa1a*, *gc*, *agxtb*, *tat*, and *tdo2a*. Notable structures are outlined with a white dotted line: cv=central vein, pv=portal vein. Scale bars, 50 microns.

Supplemental Figure 3. Related to Figure 2

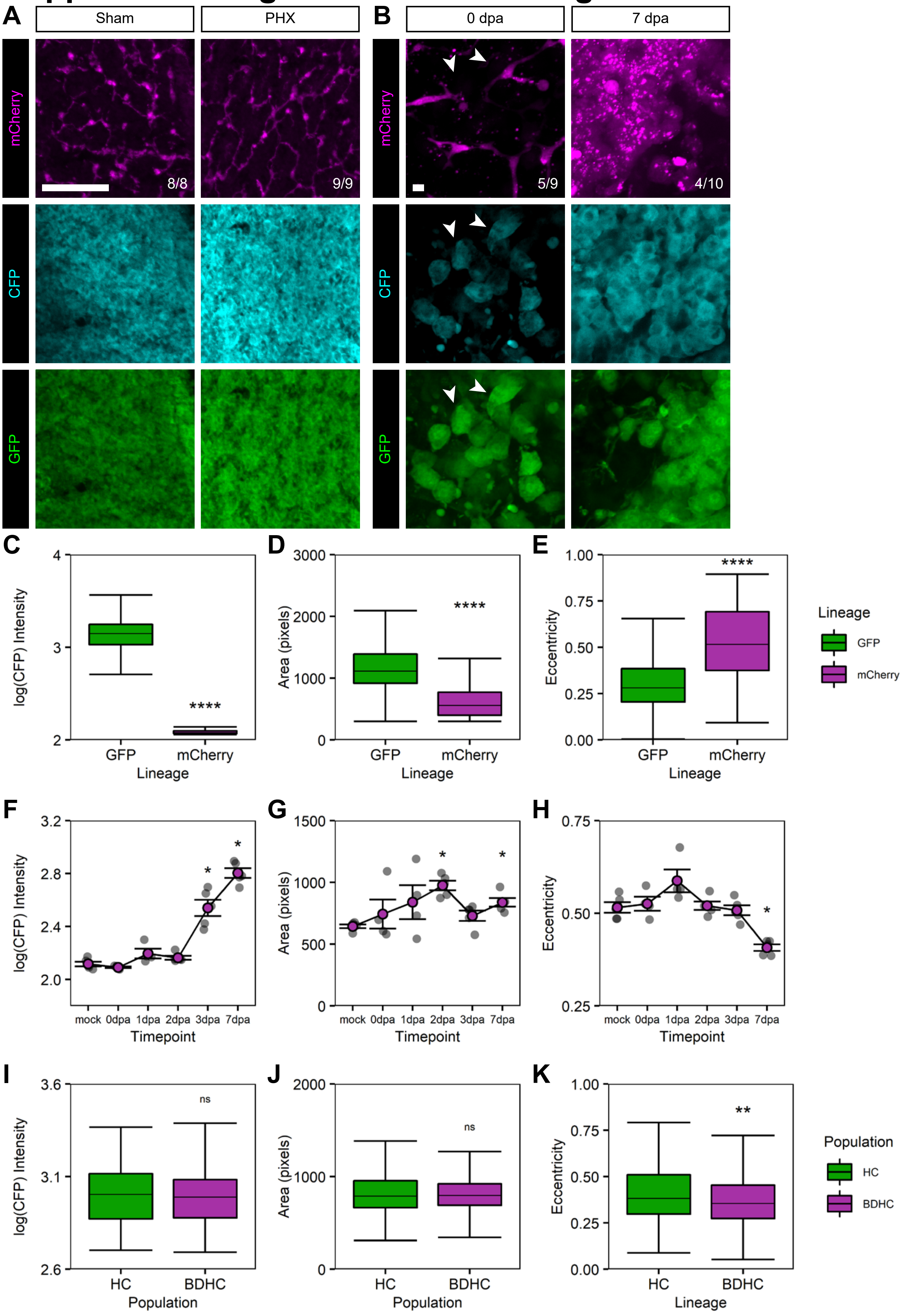

Supplemental Figure 3: Related to Figure 2

(A) Immunofluorescence showing mCherry (magenta, marking biliary and biliary-derived cells), GFP (green, marking hepatocyte-derived cells) and CFP (cyan, hepatocyte identity) signal in the adult liver for animals regenerating from surgical injury. Sham=surgical control (n=8) and PHX=partial hepatectomy (n=9). Number of animals resembling the representative image are in white in the lower right corner of each image. Scale bars, 50 microns.

(B) Live imaging time course of vibratome sections showing mCherry (magenta) and CFP (cyan) signal for adult livers regenerating after MTZ-induced hepatocyte ablation. Time points displayed are 0 dpa (n=9) and 7 dpa (n=10). At 0 dpa, white arrowheads point to GFP+ hepatocytes that survived the ablation. At 7 dpa, these hepatocytes have contributed to regeneration. Number of animals resembling the representative image are in white in the lower right corner of each image. Scale bars, 5 microns.

(C-E) Box plot of the GFP (green) and mCherry (magenta) cells in mock treated animals for (C) log(CFP) intensity, (D) area and (E) eccentricity. The box plots depict the minimum and maximum values (whiskers), the upper and lower quartiles, and the median. GFP+ cells have higher CFP intensity, are larger, and more circular than mCherry+ cells. Significance was determined using the Wilcoxon Rank Sum test. ****$p<0.0001$.

(F-H) Line graph of the mCherry (magenta) cells over time for (F) log(CFP) intensity, (G) area and (H) eccentricity. Gray dots mark the average value for an animal, magenta-filled dot marks the average of the animal values. Error bars represent standard error of the mean. mCherry+ cells gain change over time to gain CFP expression, increase in size,

and become more circular. Significance was determined using the Wilcoxon Rank Sum test, and p-values were adjusted for multiple hypothesis testing using a Bonferroni correction. *$p<0.05$.

(I-K) Box plot of the GFP (green) and mCherry (magenta) hepatocytes in 7 dpa animals for (I) log(CFP) intensity, (J) area and (K) eccentricity. The box plots depict the minimum and maximum values (whiskers), the upper and lower quartiles, and the median. On the basis of CFP intensity and area, mCherry+ hepatocytes are indistinguishable from GFP+ hepatocytes. mCherry+ hepatocytes are even more circular than GFP+ hepatocytes. Significance was determined using the Wilcoxon Rank Sum test. **$p<0.01$, n.s., not significant.

Supplemental Figure 4. Related to Figure 2

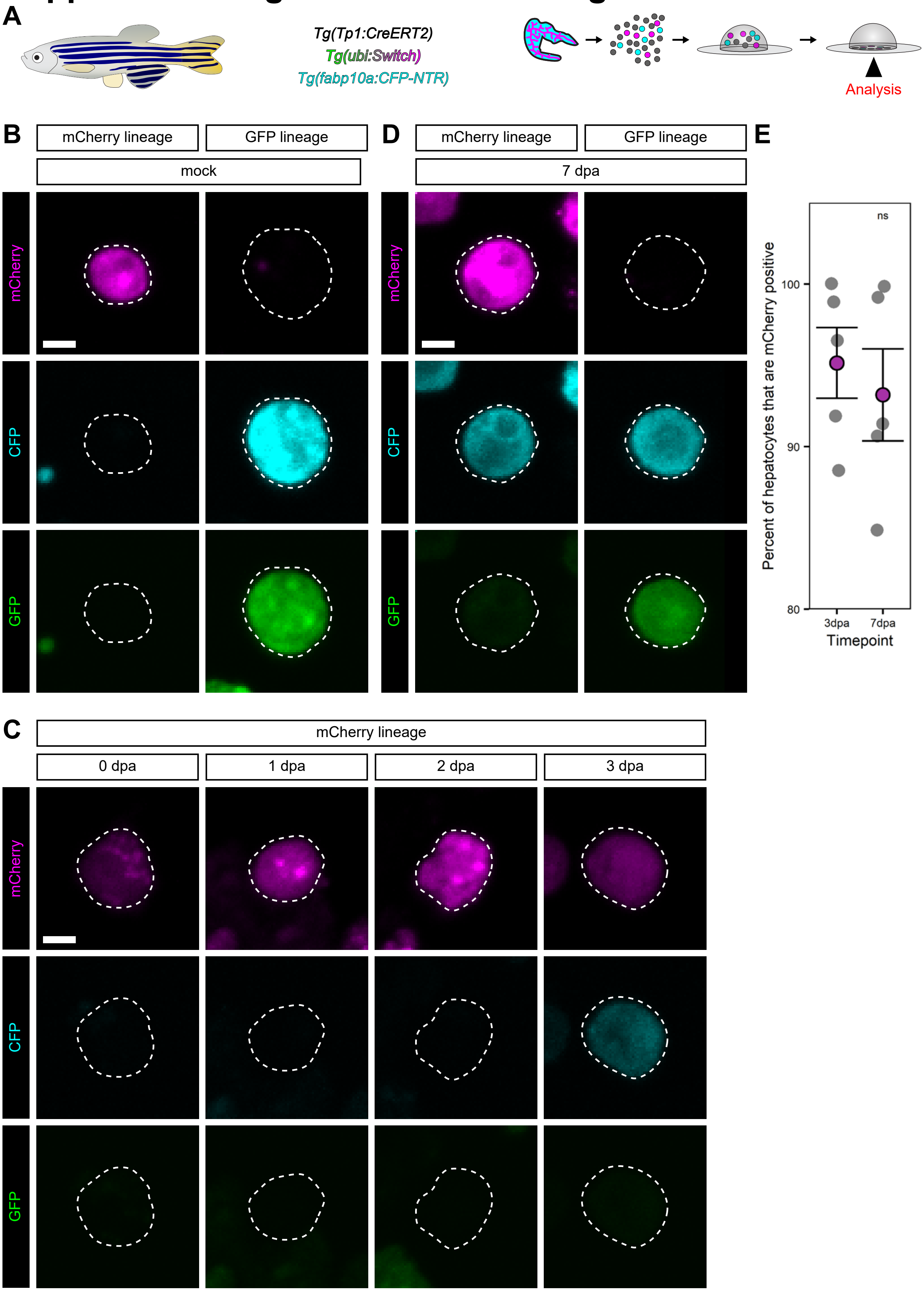

Supplemental Figure 4: Related to Figure 2

(A) Schematic indicating that zebrafish livers were isolated and dissociated into a single cell suspension. Cells were plated onto a glass slide and immediately imaged.

(B-D) Live imaging on dissociated cells showing mCherry (magenta), GFP (green) and CFP (cyan) signal for animals regenerating from hepatocyte ablation. Time points shown are (B) mock (n=5), (C) 0 dpa (n=4), 1 dpa (n=4), 2 dpa (n=5) and 3 dpa (n=5), and (D) 7 dpa (n=5). White dashed lines mark the cell boundary. mCherry+ cells acquire CFP expression at 3 dpa. Scale bars, 5 microns.

(E) Dot plot of the percentages of hepatocytes that are mCherry+. Gray dots mark the average value for an animal, magenta-filled dot marks the average of the animal values for 3 dpa (n=5) and 7 dpa (n=5). Error bars represent standard error of the mean. Significance was determined using the Wilcoxon Rank Sum test. n.s., not significant.

Supplemental Figure 5. Related to Figure 3

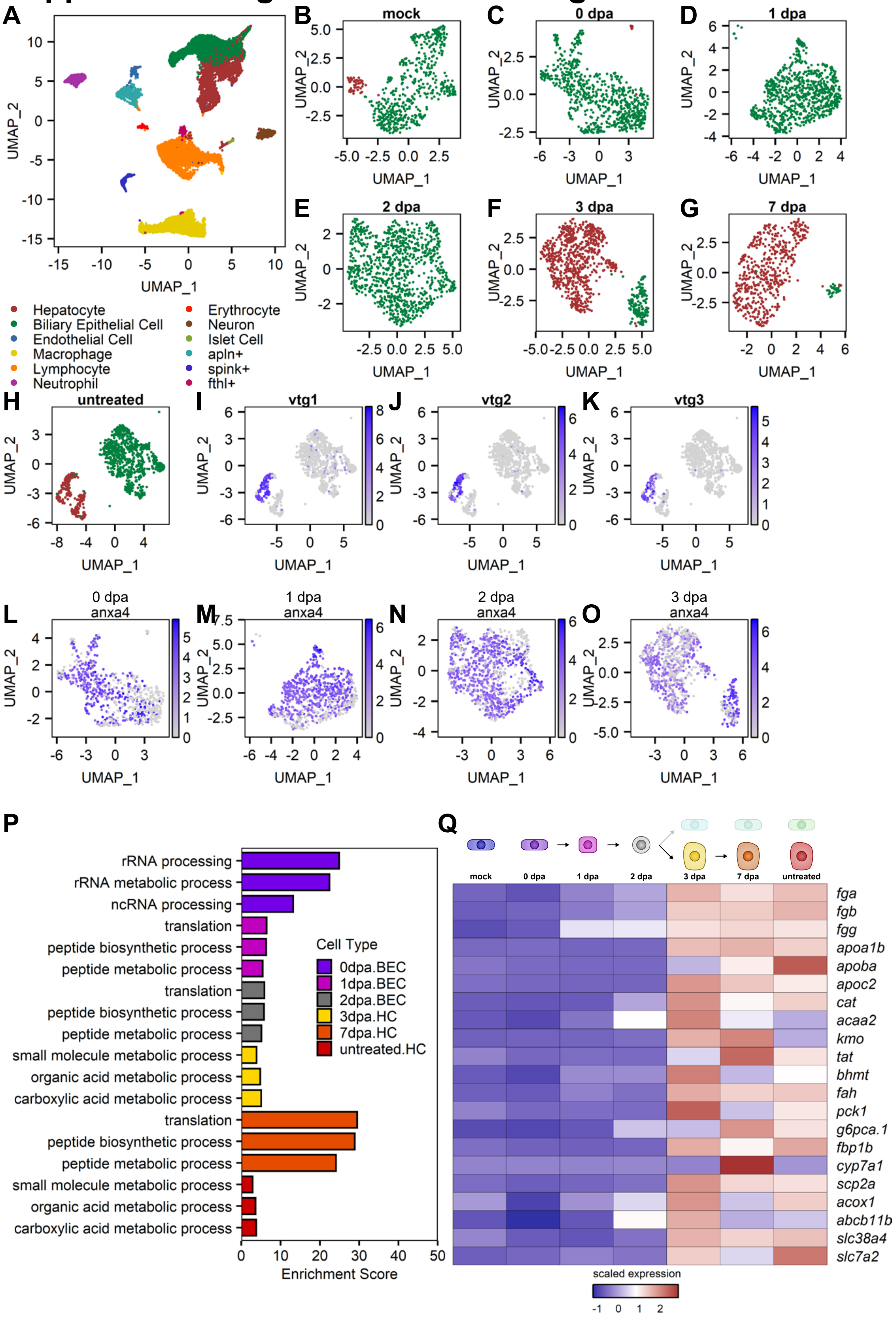

Supplemental Figure 5: Related to Figure 3

(A) UMAP plots of the cells during regeneration from hepatocyte ablation, with 12 clusters identified.

(B-H) UMAP plots of the hepatocytes (red) and BECs (green) from individual time points, including (B) mock, (C) 0 dpa, (D) 1 dpa, (E) 2 dpa, (F) 3 dpa, (G) 7 dpa, and (H) untreated. Hepatocytes reemerge in the dataset at 3 dpa.

(I-J) Feature plots on untreated hepatocytes and BECs for (I) *vtg1*, (J) *vtg2*, and (K) *vtg3* reveal presumed hepatocytes from female livers. The color key from gray to blue indicates low to high expression levels, respectively.

(L-O) Feature plots for *anxa4* at (L) 0 dpa, (M) 1 dpa, (N) 2 dpa, and (O) 3 dpa. The color key from gray to blue indicates low to high expression levels, respectively.

(P) Bar graph for the top three GO terms for each selected cell state cluster. The length of the bar is the level of enrichment for that term in that cluster. The bars are colored by cluster.

(Q) Heatmap of the average expression in selected cell states for each gene along the hepatocyte branch. The scaled expression values are average expression values that have been normalized to the minimum and maximum values in each row. The color key from blue to red indicates low to high scaled expression levels, respectively. Hepatocyte functions are restored at 3 dpa.

Supplemental Figure 6. Related to Figure 3

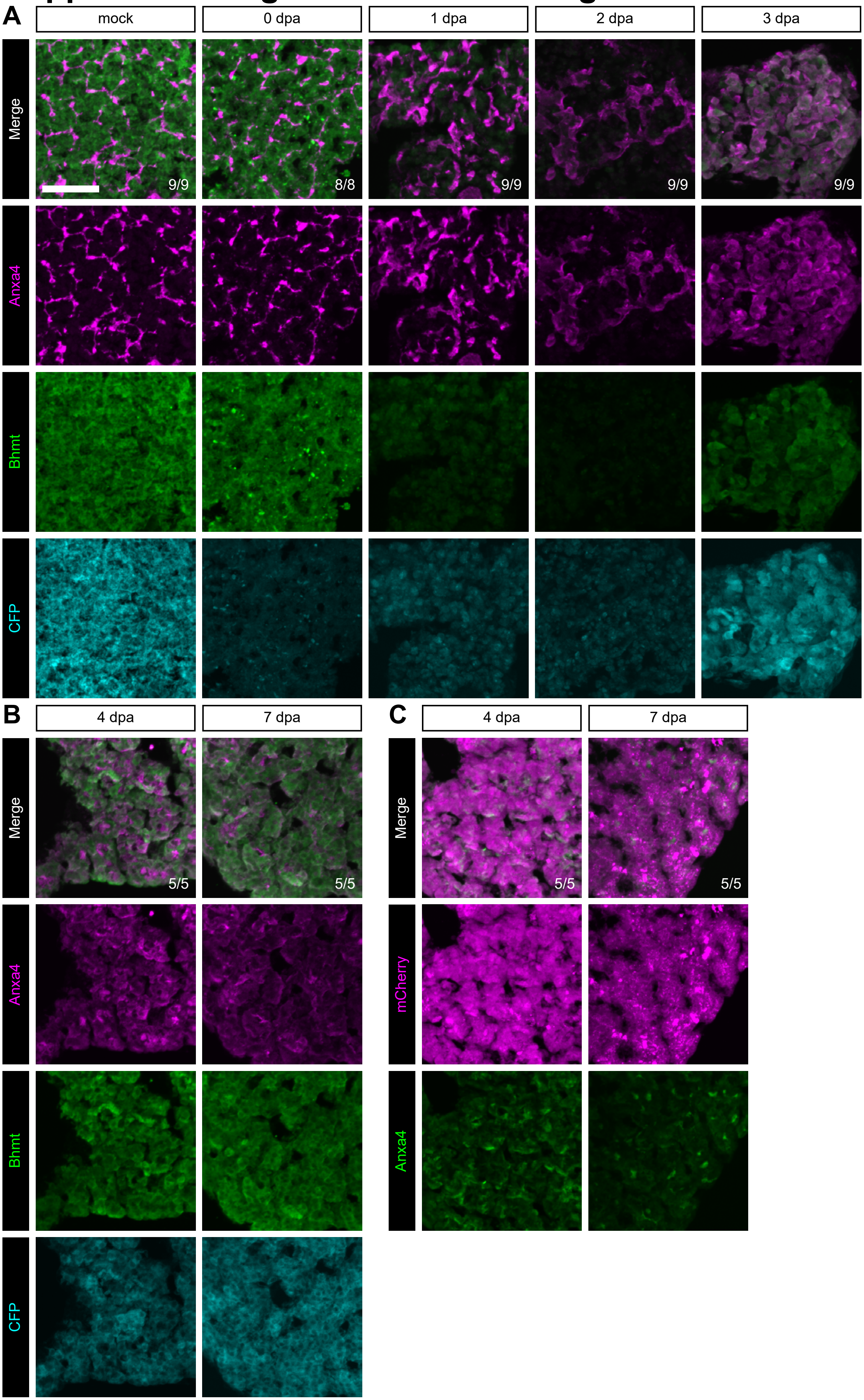

Supplemental Figure 6: Related to Figure 3

(A-B) Immunofluorescence showing Anxa4 (magenta), Bhmt (green), and CFP (cyan) signal for animals regenerating from hepatocyte ablation. Time points shown are (A) mock (n=9), 0 dpa (n=8), 1 dpa (n=9), 2 dpa (n=9), and 3 dpa (n=9), and (B) 4 dpa (n=5) and 7 dpa (n=5). Bhmt and CFP signal have reemerged by 3 dpa. Number of animals resembling the representative image are in white in the lower right corner of each image. Scale bars, 50 microns.

(C) Immunofluorescence showing mCherry (magenta) and Anxa4 (green) signal as markers for BEC origin and BEC identity, respectively, in adult livers in animals regenerating from hepatocyte ablation. Time points shown are 4 dpa (n=5) and 7 dpa (n=5). Number of animals resembling the representative image are in white in the lower right corner of each image. Scale bars, 50 microns.

Supplemental Figure 7. Related to Figures 4, 5 and 6

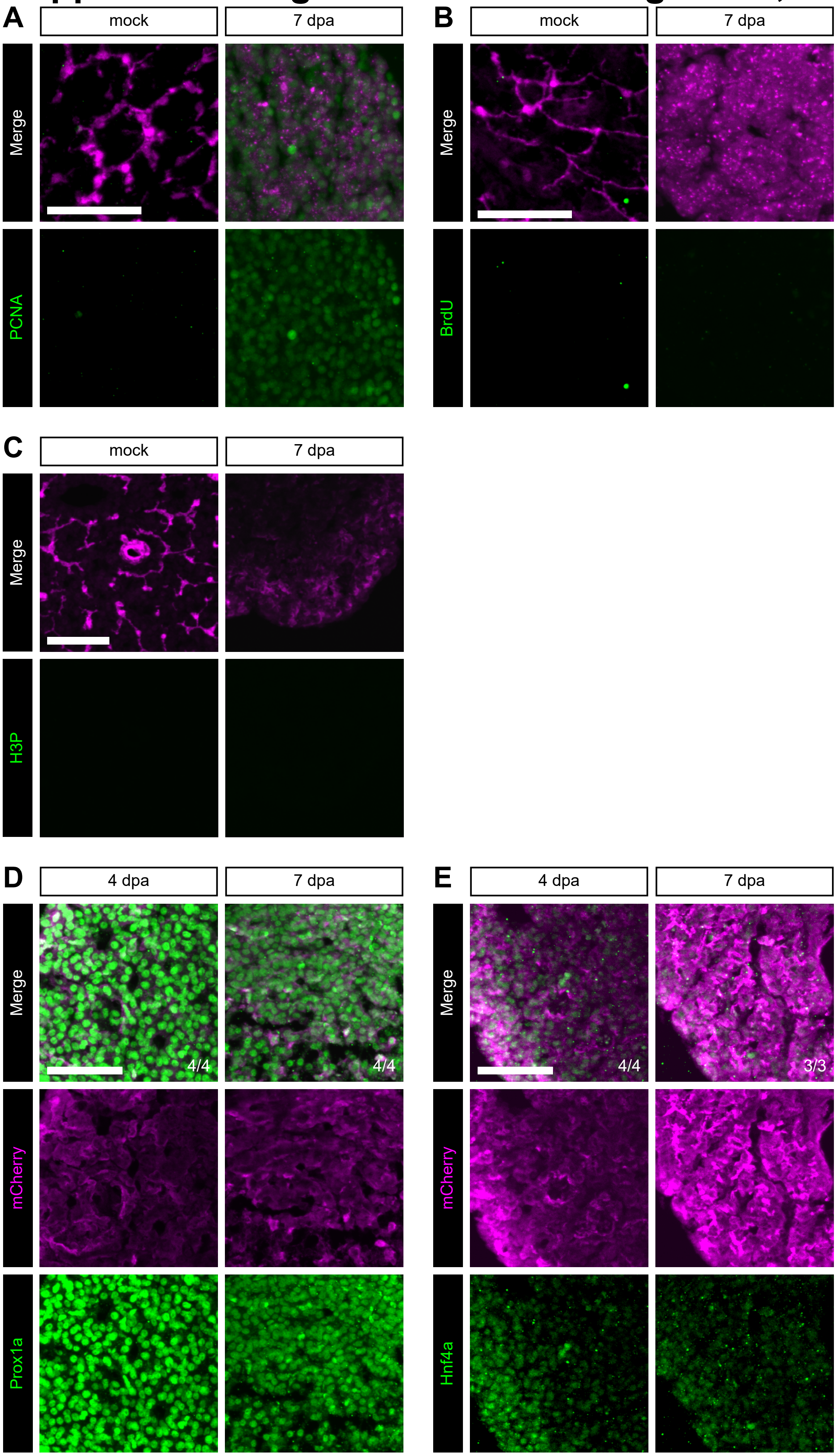

Supplemental Figure 7: Related to Figures 4, 5 and 6

(A-C) Immunofluorescence in adult liver showing markers of biliary origin (magenta) and proliferation/cell cycling (green); (A) mCherry (magenta) and PCNA (green), (B) mCherry (magenta) and BrdU (green), or (C) Anxa4 (magenta) and H3P (green) signal for animals regenerating from hepatocyte ablation. Time points shown are PCNA: mock (n=8) and 7 dpa (n=5), BrdU: mock (n=8) and 7 dpa (n=4), and H3P: mock (n=9) and 7 dpa (n=5). Scale bars, 50 microns.

(D-E) Immunofluorescence in adult liver showing markers of biliary origin (magenta) and transcription factor levels (green); (D) mCherry (magenta) and Prox1a (green) or (E) mCherry (magenta) and Hnf4a (green) signal for animals regenerating from hepatocyte ablation. Time points shown are Prox1a: 4 dpa (n=4) and 7 dpa (n=4) and Hnf4a: 4 dpa (n=4) and 7 dpa (n=3). Number of animals resembling the representative image are in white in the lower right corner of each image. Scale bars, 50 microns.

Supplemental Figure 8. Related to Figures 4 and 5

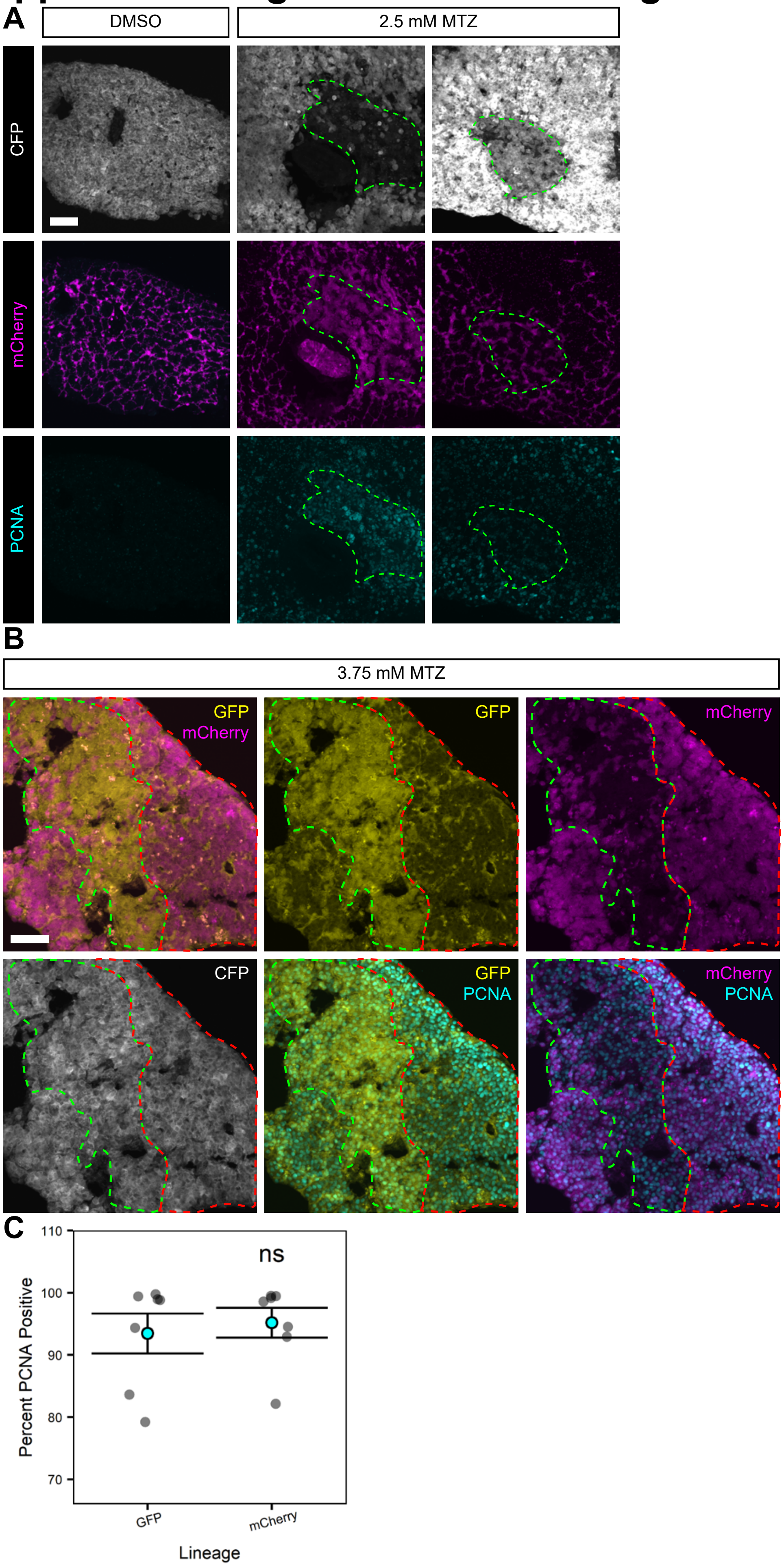

Supplemental Figure 8: Related to Figures 4 and 5

(A) Immunofluorescence in adult liver showing signal for CFP expression (gray), mCherry (magenta) and PCNA (cyan). Samples shown are DMSO (n=4) and 2.5 mM MTZ (n=2) at 3 dpa. Green dotted line outlines an ablated area. Two examples are shown for 2.5 mM MTZ group. Biliary epithelial cells inside and outside the area have different morphologies. Scale bars, 50 microns.

(B) Immunofluorescence in adult liver showing signal for CFP expression (gray), GFP (yellow), mCherry (magenta), and PCNA (cyan). Samples shown are animals treated with 3.75 mM MTZ (n=7) at 3 dpa. Green dotted line outlines cells of hepatocyte origin and the red dotted line outlines cells of a biliary origin. Scale bars, 50 microns.

(C) Dot plot of the percentages of nuclei positive for PCNA in either GFP+ or mCherry+ cells. Gray dots mark the average value for an animal, cyan-filled dot marks the average of the animal values. Error bars represent standard error of the mean. There is no difference in the proliferative index of GFP+ and mCherry+ cells. Significance was determined using the Wilcoxon Rank Sum test. n.s., not significant.

Supplemental Figure 9. Related to Figures 4 and 5

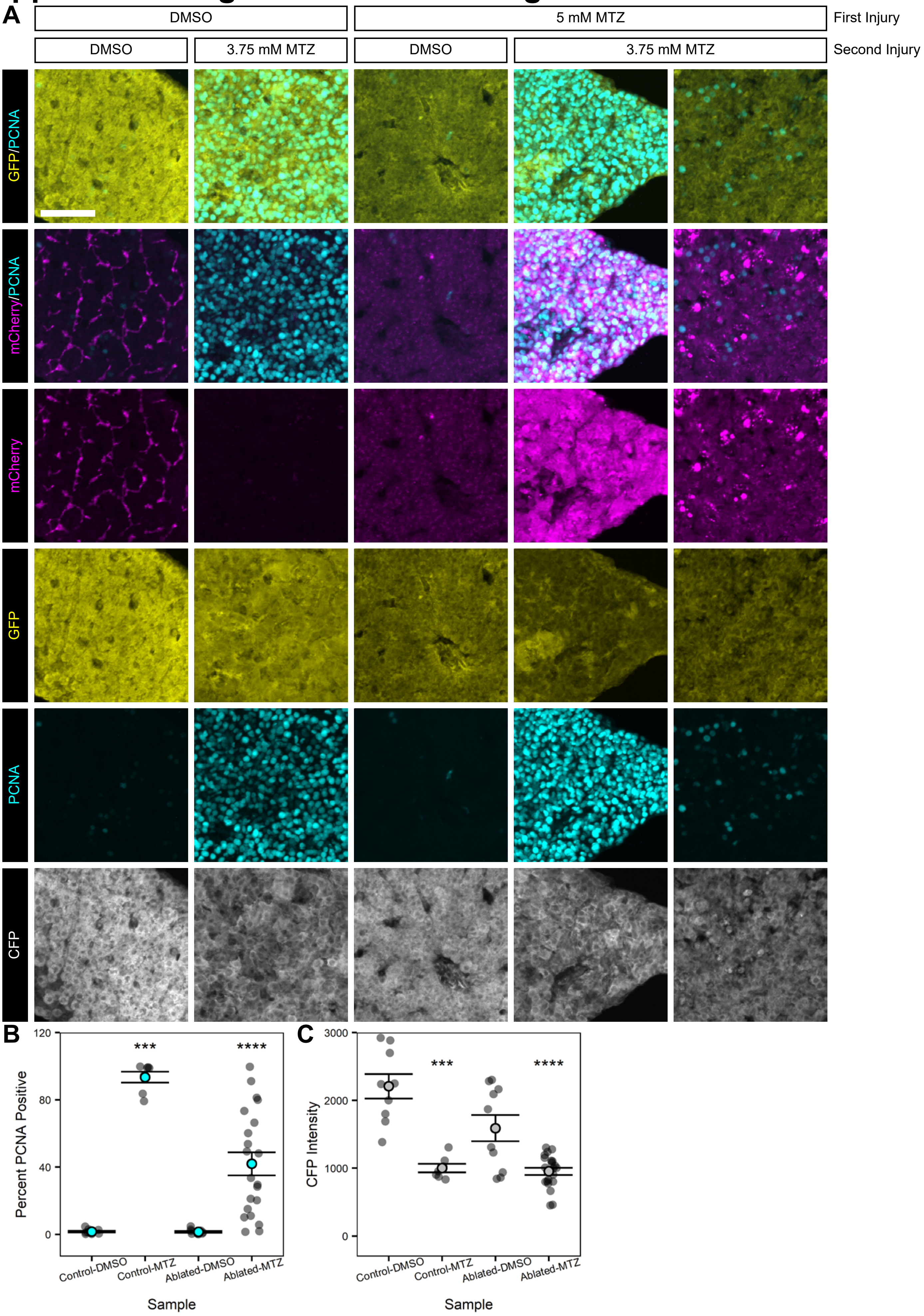

Supplemental Figure 9: Related to Figures 4 and 5

(A) Immunofluorescence in adult liver showing signal for CFP expression (gray), GFP (yellow), mCherry (magenta), and PCNA (cyan). Samples shown are animals subjected to a first injury, allowed to recover, and then subjected to a second injury. Samples are DMSO/DMSO (n=9), DMSO/3.75 mM MTZ (n=13), 5 mM MTZ/DMSO (n=10), and 5 mM MTZ/3.75 mM MTZ (n=21) at 3 dpa following the second injury. Two examples are shown for the 5 mM MTZ/3.75 mM MTZ group demonstrating the range of phenotypes. Scale bars, 50 microns.

(B) Dot plot of the percentages of nuclei positive for PCNA. Gray dots mark the average value for an animal, cyan-filled dot marks the average of the animal values. Error bars represent standard error of the mean. Significance was determined using the Wilcoxon Rank Sum test, and p-values were adjusted for multiple hypothesis testing using a Bonferroni correction. ***$p<0.001$, ****$p<0.0001$.

(C) Dot plot of the nuclear CFP intensity values. Light gray dots mark the average value for an animal, solid gray-filled dot marks the average of the animal values. Error bars represent standard error of the mean. Significance was determined using the Wilcoxon Rank Sum test, and p-values were adjusted for multiple hypothesis testing using a Bonferroni correction. ***$p<0.001$, ****$p<0.0001$.

Supplemental Figure 10. Related to Figure 7

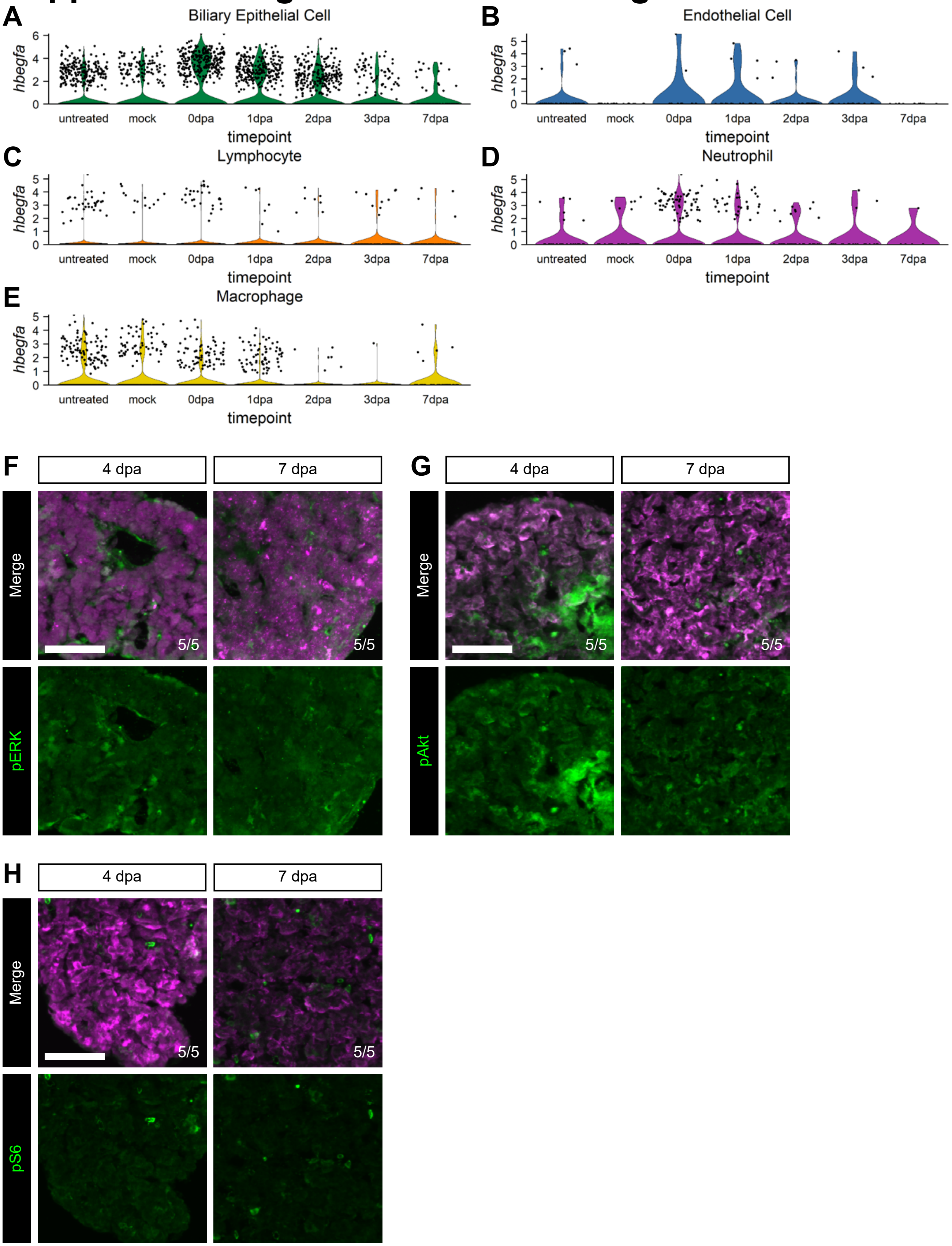

Supplemental Figure 10: Related to Figure 7

(A-E) Violin plots of single-cell gene expression for *hbegfa* for (A) biliary epithelial cells, (B) endothelial cells, (C) lymphocytes, (D) neutrophils, and (E) macrophages.

(F-H) Immunofluorescence in adult livers showing (F) mCherry (magenta) and pERK (green), (G) Anxa4 (magenta) and pAkt (green), or (H) Anxa4 (magenta) and pS6 (green) signal for animals regenerating from hepatocyte ablation. Time points shown are pERK: 4 dpa (n=5) and 7 dpa (n=5), pAkt: 4 dpa (n=5) and 7 dpa (n=5), and pS6: 4 dpa (n=5) and 7 dpa (n=5). ERK, Akt and mTOR signaling are increased in the course of regeneration. Number of animals resembling the representative image are in white in the lower right corner of each image. Scale bars, 50 microns.

Supplemental Figure 11. Related to Figure 8

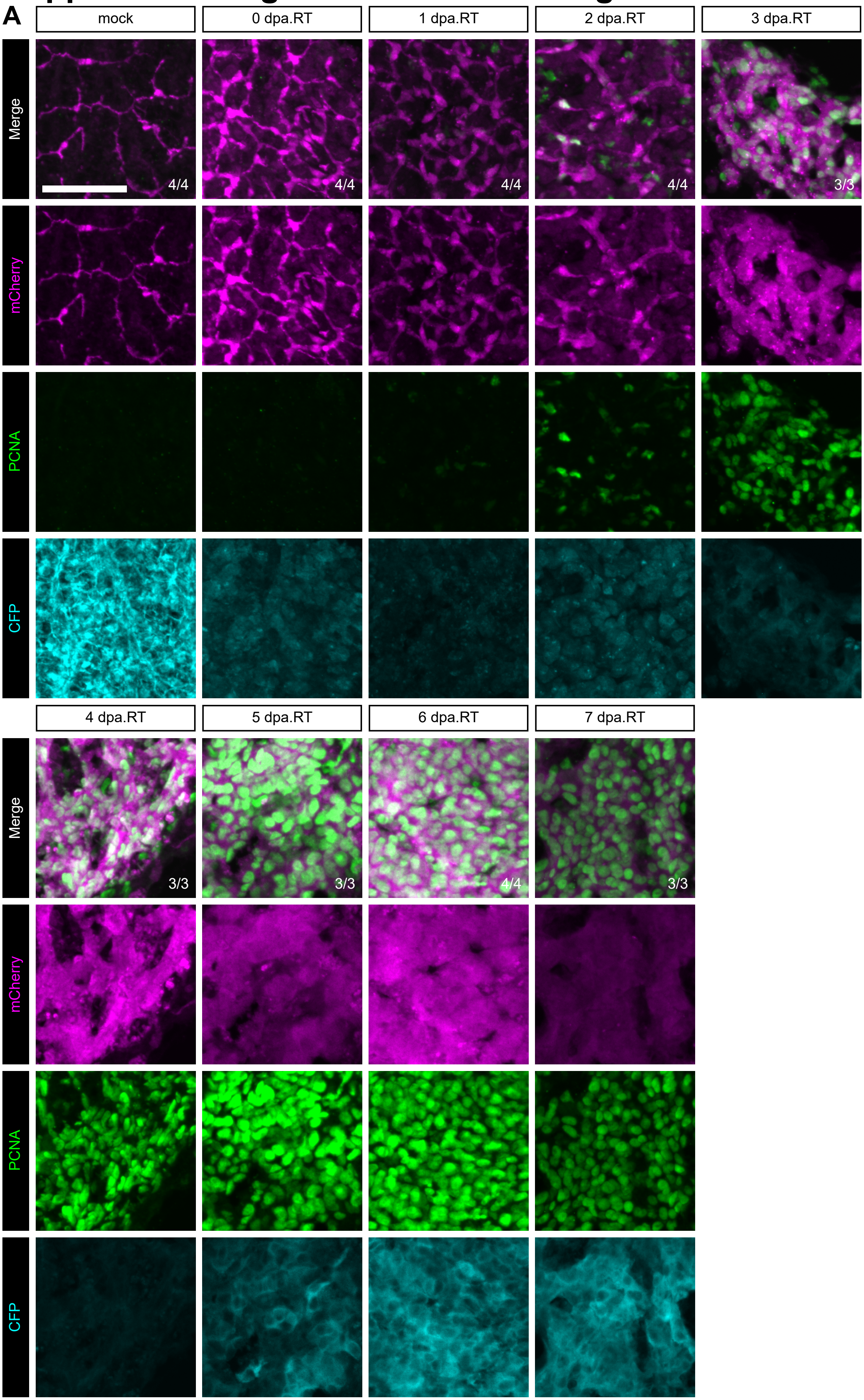

Supplemental Figure 11: Related to Figure 8

(A) Immunofluorescence showing mCherry (magenta), PCNA (green), and CFP (cyan) signal for animals regenerating from hepatocyte ablation at room temperature. Time points shown are mock (n=4), 0 dpa.RT (n=4), 1 dpa.RT (n=4), 2 dpa.RT (n=4), 3 dpa.RT (n=3), 4 dpa.RT (n=3), 5 dpa.RT (n=3), 6 dpa.RT (n=4), and 7 dpa.RT (n=3). Regeneration at room temperature proceeds with slower kinetics as compared to regeneration at 28.5°C. Number of animals resembling the representative image are in white in the lower right corner of each image. Scale bars, 50 microns.

Supplemental Figure 12. Related to Figure 8

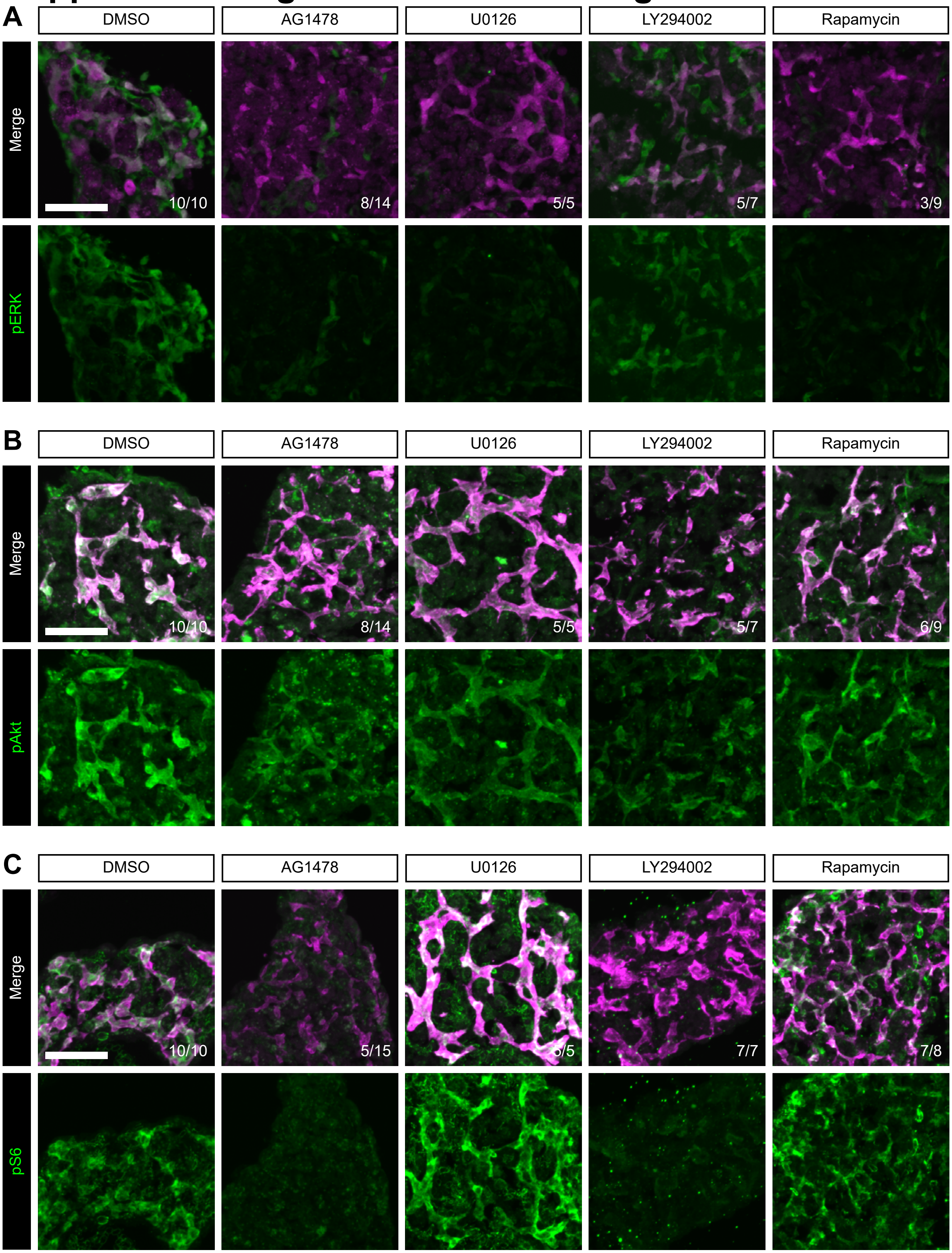

Supplemental Figure 12: Related to Figure 8

(A-C) Immunofluorescence showing (A) mCherry (magenta) and pERK (green), (B) Anxa4 (magenta) and pAkt (green), or (C) Anxa4 (magenta) and pS6 (green) signal for regenerating animals at 3dpa.RT after chemical treatments. Samples shown are pERK: DMSO (n=10), AG1478 (n=14), U0126 (n=5), LY294002 (n=7) and Rapamycin (n=9), pAkt: DMSO (n=10), AG1478 (n=14), U0126 (n=5), LY294002 (n=7) and Rapamycin (n=9), and pS6: DMSO (n=10), AG1478 (n=15), U0126 (n=5), LY294002 (n=7) and Rapamycin (n=8). Number of animals resembling the representative image are in white in the lower right corner of each image. Scale bars, 50 microns.

Supplemental Figure 13. Related to Figure 8

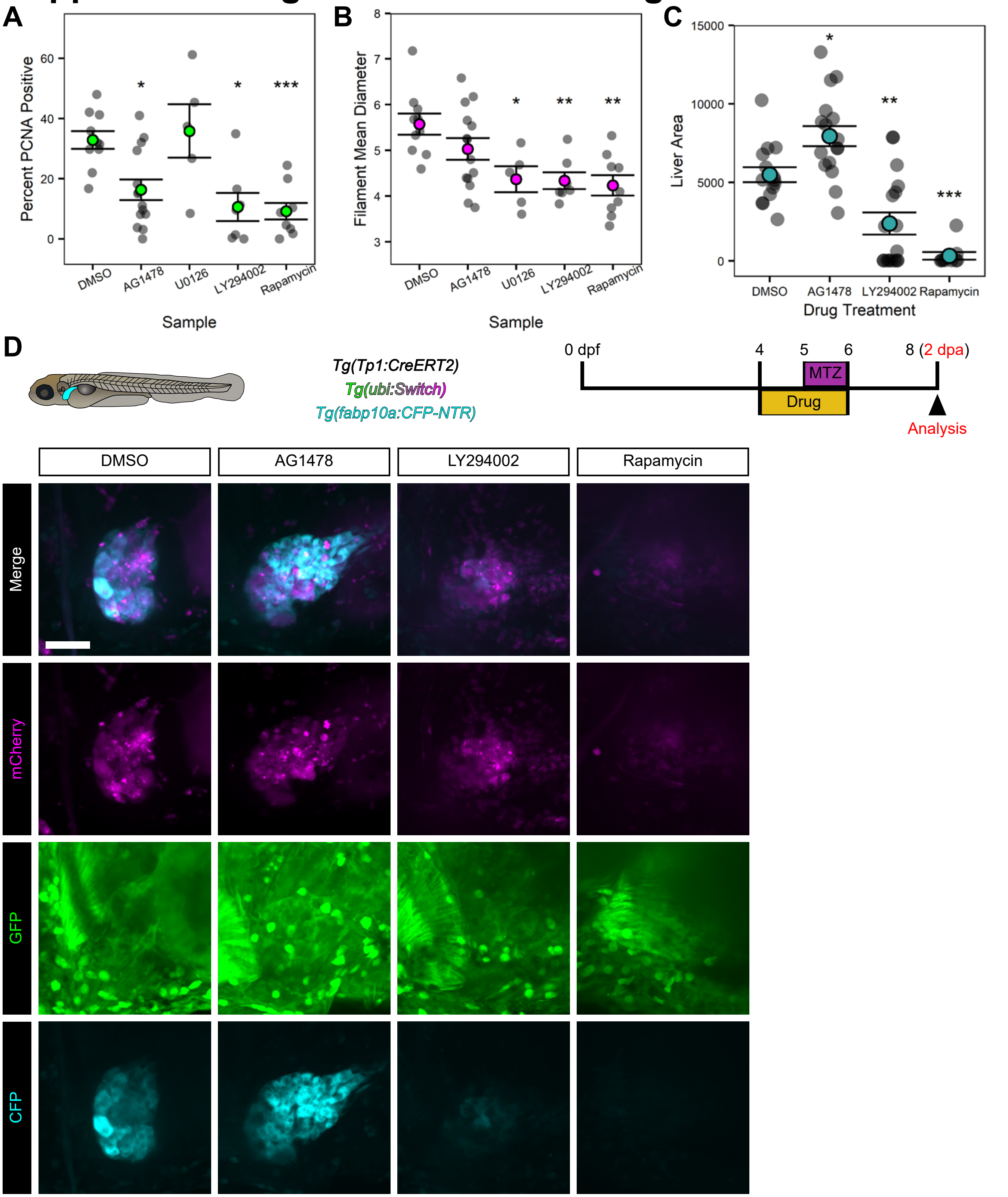

Supplemental Figure 13: Related to Figure 8

(A) Dot plot of the percentages of nuclei positive for PCNA. Gray dots mark the average value for an animal, green-filled dot marks the average of the animal values. Error bars represent standard error of the mean. Significance was determined using the Wilcoxon Rank Sum test, and p-values were adjusted for multiple hypothesis testing using a Bonferroni correction. *p<0.05, ***p<0.001.

(B) Dot plot of the average filament mean diameter for mCherry+ cells. Gray dots mark the average value for an animal, magenta-filled dot marks the average of the animal values. Error bars represent standard error of the mean. Significance was determined using the Wilcoxon Rank Sum test, and p-values were adjusted for multiple hypothesis testing using a Bonferroni correction. *p<0.05, **p<0.01.

(C) Dot plot of the liver areas for larval zebrafish. Gray dots mark the average value for an animal, cyan-filled dot marks the average of the animal values. Error bars represent standard error of the mean. Significance was determined using the Wilcoxon Rank Sum test, and p-values were adjusted for multiple hypothesis testing using a Bonferroni correction. *p<0.05, **p<0.01, ***p<0.001.

(D) Live imaging time course showing mCherry (magenta), GFP (green), and CFP (cyan) signal for larval zebrafish livers regenerating after MTZ-induced hepatocyte ablation and treatment with a chemical inhibitor. Samples include DMSO (n=15), AG1478 (n=17), LY294002 (n=17), and Rapamycin (n=9). Scale bars, 50 microns.

Supplemental Figure 14. Related to Figure 8

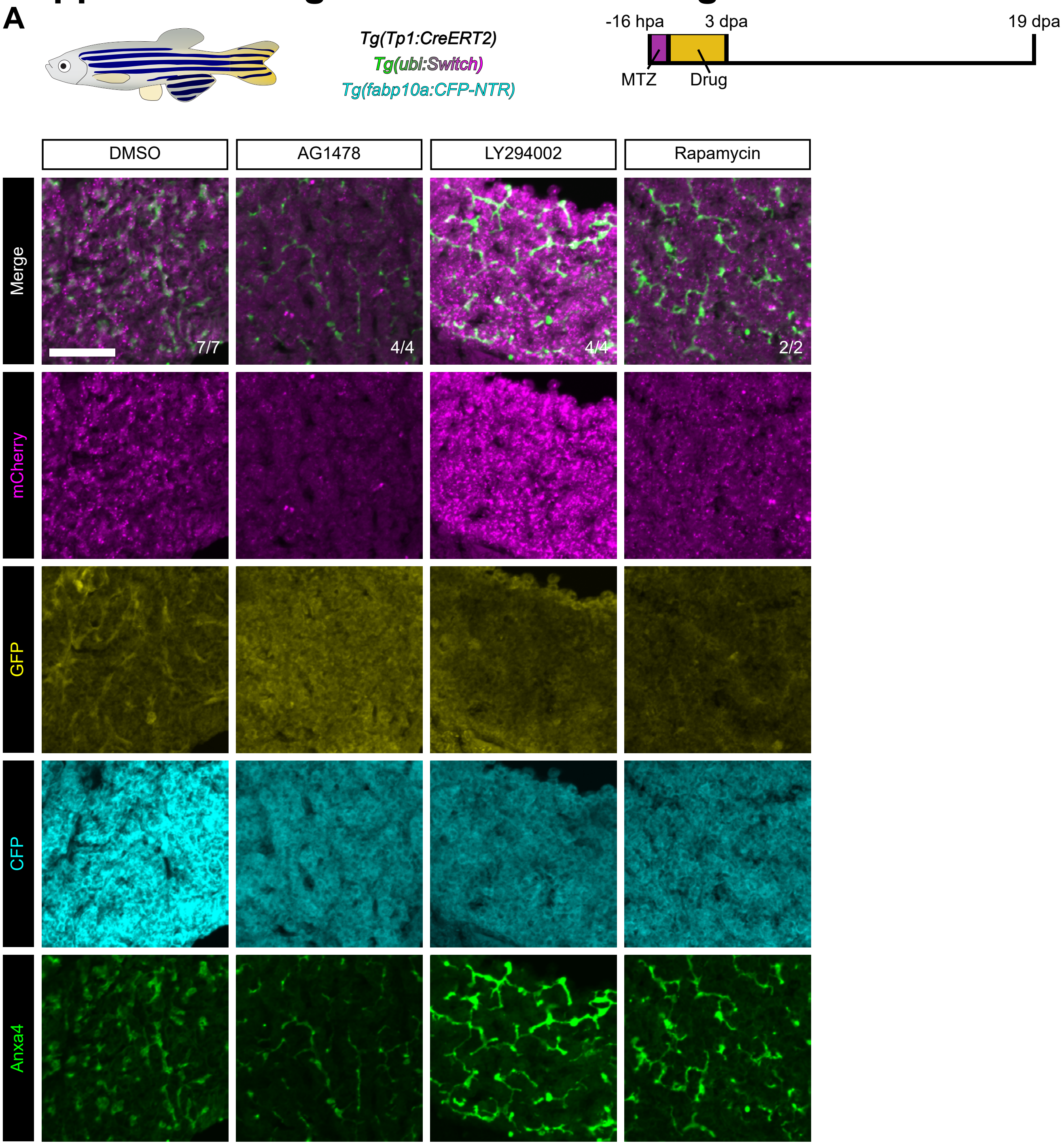

Supplemental Figure 14: Related to Figure 8

(A) Immunofluorescence in adult liver showing signal for mCherry (magenta), GFP (yellow), CFP (cyan), and Anxa4 (green). Samples shown are animals subjected to an injury and allowed to recover until 19 dpa. Samples are DMSO (n=7), AG1478 (n=4), LY294002 (n=4), and Rapamycin (n=2). Scale bars, 50 microns.

Supplemental Figure 15. Related to Figure 8

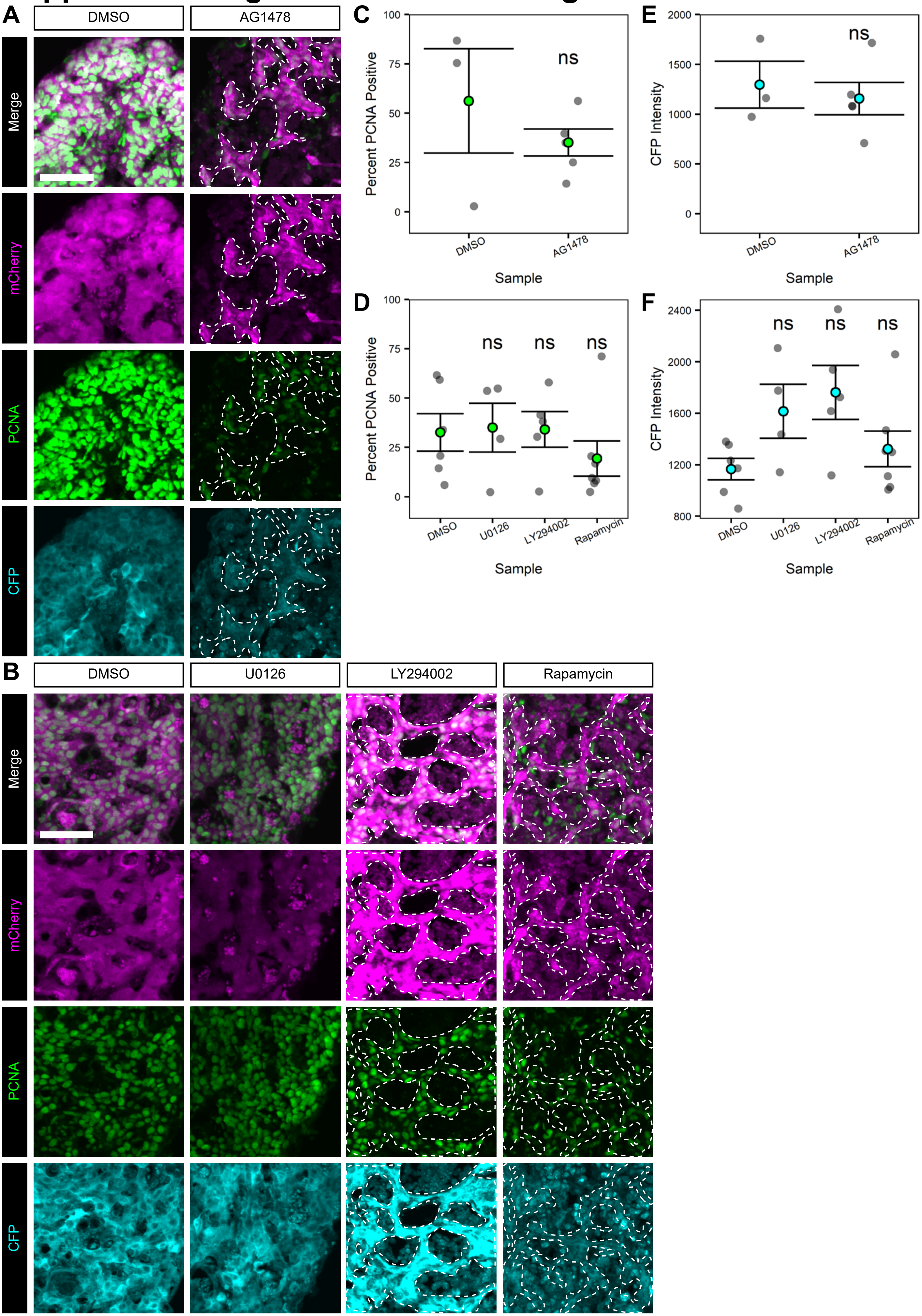

Supplemental Figure 15: Related to Figure 8

(A-B) Immunofluorescence showing mCherry (magenta), PCNA (green), and CFP (cyan) signal for animals regenerating from hepatocyte ablation at (A) 5dpa.RT or (B) 6dpa.RT after chemical treatments. Samples shown are 5 dpa.RT: DMSO (n=3) and AG1478 (n=5), and 6 dpa.RT: DMSO (n=6), U0126 (n=4), LY294002 (n=5), and Rapamycin (n=7). White dotted lines outline cells of interest. Scale bars, 50 microns.

(C-D) Dot plot of the percentages of nuclei positive for PCNA, for (C) 5 dpa.RT and (D) 6 dpa.RT. Gray dots mark the average value for an animal, green-filled dot marks the average of the animal values. Error bars represent standard error of the mean. Significance was determined using the Wilcoxon Rank Sum test. n.s., not significant.

(E-F) Dot plot of the nuclear CFP intensity values, for (E) 5 dpa.RT and (F) 6 dpa.RT. Gray dots mark the average value for an animal, cyan-filled dot marks the average of the animal values. Error bars represent standard error of the mean. Significance was determined using the Wilcoxon Rank Sum test, and p-values were adjusted for multiple hypothesis testing using a Bonferroni correction. n.s., not significant.
